# NIN-like protein7 and PROTEOLYSIS6 functional interaction enhances tolerance to sucrose, ABA, and submergence

**DOI:** 10.1093/plphys/kiab382

**Published:** 2021-08-10

**Authors:** Mari-Cruz Castillo, Álvaro Costa-Broseta, Beatriz Gayubas, José León

**Affiliations:** Instituto de Biología Molecular y Celular de Plantas (Consejo Superior de Investigaciones Científicas–Universidad Politécnica de Valencia), Valencia 46022, Spain

## Abstract

Nitrate (NO_3_) assimilation and signaling regulate plant growth through the relevant function of the transcription factor NIN-like Protein7 (NLP7). NO_3_ is also the main source for plants to produce nitric oxide (NO), which regulates growth and stress responses. NO-mediated regulation requires efficient sensing via the PROTEOLYSIS6 (PRT6)-mediated proteasome-triggered degradation of group VII of ethylene response transcription factors through the Cys/Arg N-degron pathway. The convergence of NO_3_ signaling and N-degron proteolysis on NO-mediated regulation remains largely unknown. Here, we investigated the functional interaction between NLP7 and PRT6 using Arabidopsis (*Arabidopsis thaliana*) double *prt6 nlp7* mutant plants as well as complementation lines overexpressing *NLP7* in different mutant genetic backgrounds. *prt6 nlp7* mutant plants displayed several potentiated *prt6* characteristic phenotypes, including slower vegetative growth, increased NO content, and diminished tolerance to abiotic stresses such as high-sucrose concentration, abscisic acid, and hypoxia–reoxygenation. Although NLP7 has an N-terminus that could be targeted by the N-degron proteolytic pathway, it was not a PRT6 substrate. The potential PRT6- and NO-regulated nucleocytoplasmic translocation of NLP7, which is likely modulated by posttranslational modifications, is proposed to act as a regulatory loop to control NO homeostasis and action.

## Teaser

Nitrate signaling and N-degron-mediated proteolysis interact to regulate plant tolerance to abiotic stresses.

## Introduction

Plant growth and development largely depend on the efficient nutrition of essential elements such as nitrogen (N), which is thus a limiting factor for crop production in agriculture (Xu et al., 2012; de Bang et al., 2021). Nitrate (NO_3_) is the more abundant and accessible inorganic N source for plant nutrition (Crawford and Glass, 1998) and ensures the biosynthesis of key molecules for life such as amino acids and nucleotides. Besides, N nutrition is key to produce other N-containing molecules among which nitric oxide (NO) has been extensively characterized as a relevant regulator of plant development and stress responses (León and Costa-Broseta, 2020). NO biosynthesis in plants has been proposed to occur through both oxidative and reductive pathways, though the reductive pathway connected to NO_3_ assimilation seems to be the main source (Astier et al., 2018). Reductive NO production in plants involves the function of enzymes of the NO_3_ assimilatory pathway, more precisely the action of cytoplasmic NAD(P)H-NO_3_ reductases (NRs; Campbell, 2001), which largely regulate NO homeostasis (Chamizo-Ampudia et al., 2017) and multiple NO-modulated stress and developmental responses. NRs catalyze mainly the reduction of NO_3_ to nitrite (Campbell, 1999), but alternatively also reduce nitrite to NO under certain conditions (Rockel et al., 2002). In Arabidopsis (*Arabidopsis thaliana*), *nia1nia2* plants carrying mutations in the two NRs, NIA1 and NIA2, produced less NO (Seligman et al., 2008; Lozano-Juste and León, 2010), whereas *NIA* overexpression increased NO content (Costa-Broseta et al., 2021). The expression of *NR/NIA* genes is activated by NO_3_ through the NIN-like protein7 (NLP7) transcription factor, which binds to the NO_3_ response cis-element on their promoters (Castaings et al., 2009; Konishi and Yanagisawa, 2013). NLP7, together with NLP6, is a master regulator of primary NO_3_ responses (Castaings et al., 2009; Konishi and Yanagisawa, 2013; Marchive et al., 2013; Yu et al., 2016; Cao et al., 2017; Zhao et al., 2018). The *nlp7* mutants display NO_3_ starvation phenotypes when NO_3_ is used as the only N source (Castaings et al., 2009). Interestingly, NLP7 is translocated and retained in the nucleus in response to NO_3_, thus activating NO_3_ uptake and assimilation as well as the expression of secondary regulator-encoding genes of the NO_3_ signaling pathway (Marchive et al., 2013). NO_3_ assimilation is widely regulated at the posttranslational level (Wang et al., 2021). NR/NIA function is regulated by multiple posttranslational modifications (PTMs) that include reversible phosphorylation (Wang et al., 2010), SUMOylation (Park et al., 2011), and ubiquitination, nitration, and S-nitrosation (Costa-Broseta et al., 2021). Among different PTMs, ubiquitination, nitration, and S-nitrosation represent an auto-regulatory mechanism that controls NO biosynthesis in Arabidopsis (Costa-Broseta et al., 2021). It has also been reported that NLP7 undergoes calcium-dependent protein kinase-mediated phosphorylation, which promotes its retention in the nucleus (Liu et al., 2017).

NO regulatory roles not only depend on its production, but also rely on the capacity of plants to sense it. An important mechanism for NO sensing in Arabidopsis is connected to the function of the N-degron proteolytic pathway that depends on the N-terminal sequence of the corresponding protein substrates. Different branches of this proteolytic pathway have been described (Dissmeyer, 2019; Holdsworth et al., 2020), but specifically one of them requires molecular oxygen (O_2_) and NO to operate. The Cys/Arg branch of the N-degron pathway acts on protein substrates containing a Cys residue right after the initial Met. Just a few substrates have been characterized for this N-degron branch including the five members of the group VII of Ethylene Response Factors (ERFVIIs), the polycomb repressive complex 2 component VERNALIZATION2 (Gibbs et al., 2011, 2018; Labandera et al., 2021), and the LITTLE ZIPPER 2 transcription factor (Weits et al., 2019). All of them are substrates of the E3 ubiquitin ligase PROTEOLYSIS6 (PRT6) that acts as a recognin of their N-degrons and polyubiquitinates them before being degraded by the proteasome. The PRT6-mediated N-degron pathway action on ERFVIIs has been reported to function as a NO-sensing mechanism in Arabidopsis (Gibbs et al., 2014; Abbas et al., 2015; Hartman et al., 2019).

NO_3_ signaling and the N-degron proteolytic pathway are functionally linked to seed development and performance (Yan et al., 2016; He et al., 2016; Zhang et al., 2018). NO_3_ reduces abscisic acid (ABA) levels during imbibition by upregulating the ABA catabolism gene *cytochrome P450 707A2* (*CYP707A2)* that control dormancy (Matakiadis et al., 2009), and this gene is also upregulated by NO (Liu et al., 2009). Multiple and diverse functional interactions between NO and ABA control plant development and stress responses (León et al., 2014). Specifically, NO regulates the ABA signaling factor ABA-insensitive 5 (*ABI5)* at the transcriptional level through the PRT6 branch of the N-degron-pathway-mediated degradation of its activators ERFVIIs (Gibbs et al., 2014, 2015), but also at the posttranslational level, through PTMs that include ubiquitination, SUMOylation, and S-nitrosation (Stone et al., 2006; Miura et al., 2009; Liu and Stone, 2014; Albertos et al., 2015). In this work, we addressed the functional interaction between NO_3_ signaling and N-degron proteolysis through the phenotypic analysis of double *prt6-1nlp7-1* mutant plants and transgenic complementation lines. We found functional linkage between PRT6 and NLP7 that potentiate several growth-related and stress-activated responses, including retarded vegetative growth and reduced tolerance to high sucrose, ABA, and submergence-triggered hypoxia. However, our findings suggest that this functional interaction was not due to NLP7 acting as a PRT6 substrate in the Cys/Arg branch of the N-degron proteolytic pathway.

## Results

### NO_3_ reductase-mediated NO production is negatively regulated by the proteasome

**Figure 1:**
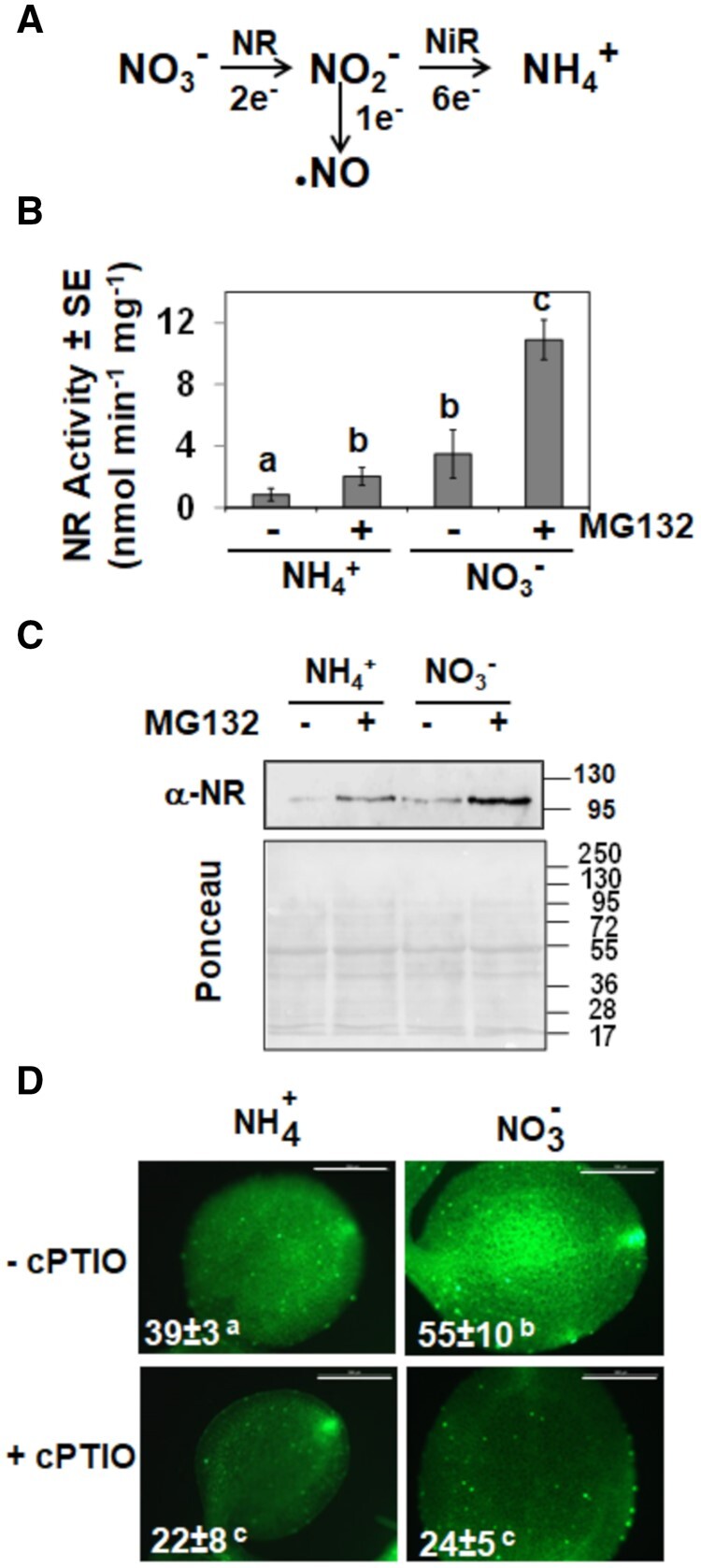
NR protein, activity, and NO content in plants grown with different N sources. A, Scheme showing different steps in the NO_3_ assimilation pathway catalyzed by NRs and nitrite reductase and the side production of NO from nitrite. B, and C, NR activity and protein, respectively, in plants grown with the indicated N source in the presence (+) or absence (−) of the proteasome inhibitor MG132. The positions of molecular mass markers (kDa) are shown to the right side of the western blot and Ponceau S-stained gel. The values are the mean ± se of three independent replicates. D, NO content in cotyledons of plants grown with the indicated N source as analyzed by staining with DAF-FM DA in the absence (−cPTIO) or presence (+cPTIO) of NO scavenger. The inserted values represent the mean ± SE of three independent replicates. The values were calculated by counting green pixels of four to six images per condition with ImageJ2/Fiji. Statistical significance was calculated by one-way ANOVA followed by Tukey’s honest significant difference (HSD) test for multiple comparisons. The letters indicate significant differences (*P* < 0.05). The scale bars represent 2 mm.

Regulatory functions exerted by NO largely depend on its biosynthesis and sensing by the plant. In *A. thaliana*, NO is mostly synthesized from NO_3_ as a side reaction of the NO_3_ assimilation pathway (Figure 1A) through a reductive process that involves the participation of two cytosolic NRs (Campbell, 2001). We analyzed the levels of NR protein and activity in plants grown with NO_3_ or ammonium (NH_4_) as the only N source. Higher activity and protein levels were detected in NO_3_-grown plants (Figure 1, B and C), and these changes corresponded with increased NO content (Figure 1D). Interestingly, we found that the application of the proteasome inhibitor MG132 led to increased NR protein and activity in both NH_4_- and NO_3_-grown plants (Figure 1, B and C), thus suggesting either NRs or an upstream activator of NRs are regulated by proteolysis through the polyubiquitination/proteasome system. We have previously reported that Arabidopsis NRs are ubiquitinated at several Lys residues (Costa-Broseta et al., 2021). On the other hand, NLP7, which is a direct activator of NR gene expression (Castaings et al., 2009; Konishi and Yanagisawa, 2013), is predicted to be potentially ubiquitinated with high scores for four Lys residues (Supplemental Table S1).

### Genetic basis of the NLP7-PRT6 functional interaction

**Figure 2:**
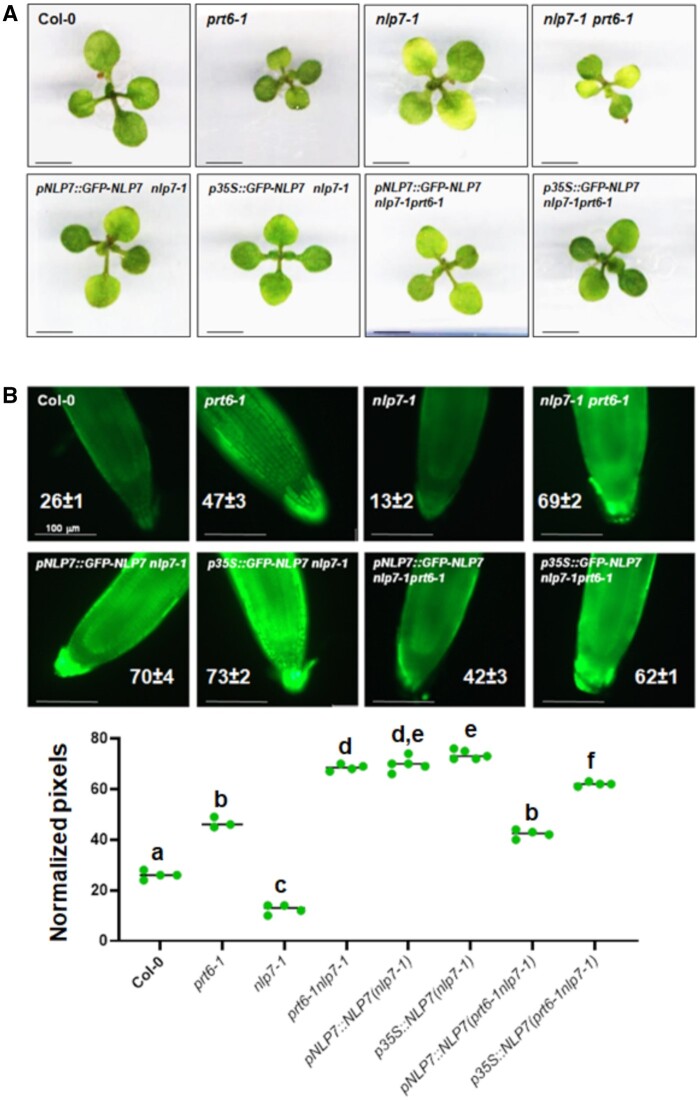
Early growth phenotype and NO content in *prt6-1nlp7-1* mutant and complementation NLP7-overexpressing lines. A, Appearance of shoots of 14-d-old seedlings of the indicated genotypes. The scale bars represent 5 mm. B, NO content in the tip of primary roots of plants of the indicated genotypes. The inserted values represent the mean ± se of three to five independent images per genotype. The values were calculated by counting green pixels of four to six images per condition with ImageJ. The scale bars represent 100 µm. Statistical significance was calculated by one-way ANOVA followed by Tukey’s HSD test for multiple comparisons. The letters indicate significant differences (*P* < 0.05).

**Figure 3:**
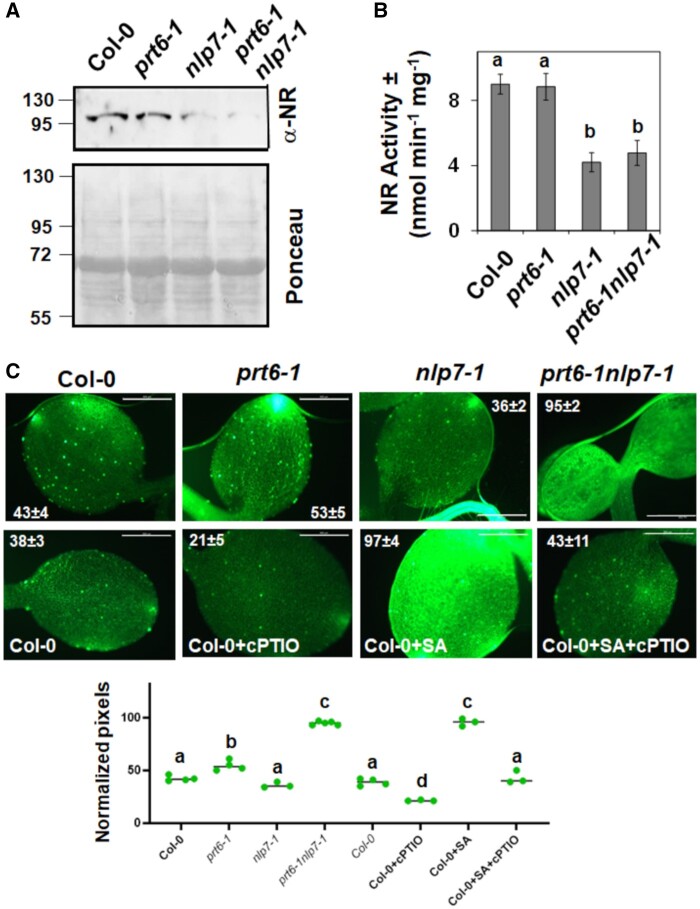
NR protein, activity, and NO content in *prt6-1*, nlp7-1, and *prt6-1nlp7-1* plants. A and B, NR protein and activity, respectively, in plants of the indicated genotypes. The positions of the molecular mass markers (kDa) are shown to the left side of the western blot and Ponceau S-stained gel. The values are the mean ± se of three independent replicates. C, NO content in cotyledons of plants was analyzed by staining with DAF-FM DA. The NO scavenger (cPTIO) and inducer SA are shown to test the specificity of the detection. The inserted values represent the mean ± se of three to five independent images per genotype. The values were calculated by counting green pixels of four to six images per condition with ImageJ2/Fiji. The scale bars represent 2 mm. Statistical significance was calculated by one-way ANOVA followed by Tukey’s HSD test for multiple comparisons. The letters indicate significant differences (*P* < 0.05).

The NLP7-activated NR-mediated biosynthesis of NO from NO_3_ is followed by sensing mechanisms that allow NO to exert diverse regulatory functions by interacting with different targets. NO can be sensed in Arabidopsis through a mechanism based on the Cys–Arg branch of the N-degron-mediated proteolysis of ERFVII transcription factors that requires the function of the recognin PRT6 (Gibbs et al., 2014). Because mutations in *NLP7 and PRT6* are likely to affect the production and sensing of NO, respectively, we crossed *prt6-1* plants to *nlp7-1* plants to generate homozygous double *prt6-1nlp7-1* plants and examined seedling phenotypes. *prt6-1* had smaller dark green seedlings relative to the wild-type, whereas *nlp7-1* seedlings were larger than *prt6-1* seedlings but chlorotic (Figure 2A). *prt6-1nlp7-1* was both small and chlorotic, indicating a combinatorial effect of the double mutation on seedling development (Figure 2A). Complementation assays of *nlp7-1 and prt6-1nlp7-1* plants performed by expressing *NLP7* under its own endogenous promoter or under a strong constitutive *35S* promoter led to plants that were all wild-type size and green (Figure 2A), thus suggesting that NLP7 can complement not only the chlorotic phenotype of the *nlp7-1* mutant, but also the small size of *prt6-1* plants. The small size phenotype typical of *prt6-1* in *nlp7-1prt6-1* seedlings was complemented by the expression of a fusion Green Fluorescent Protein (GFP)–NLP7 protein under its endogenous promoter (Figure 2A). The results suggest that GFP–NLP7 protein was more stable than native NLP7, and thus there are higher actual levels of NLP7 protein than in *prt6-1* plants. These data support the existence of functional interactions between NLP7 and PRT6. It is noteworthy that although *prt6-1 and nlp7-1* roots contained more and less NO, respectively, than in wild-type roots, the roots of double *prt6-1nlp7-1* plants contained significantly more NO than wild-type and *prt6-1* plants (Figure 2B). Besides, the expression of *NLP7* under its endogenous or *35S* promoter largely increased the NO content in the context of the *nlp7-1* background (Figure 2B). In turn, *NLP7* overexpression was not able to increase the NO content over the already high levels detected in *prt6-1nlp7-1* plants (Figure 2B). Because NLP7 activates NR-encoding genes, we expected that *nlp7-1* plants contained less NR protein and activity than Col-0 plants. Figure 3A shows that while there were no alterations in NR protein and activity levels detected in *prt6-1* plants, *nlp7-1 and prt6-1nlp7-1* plants indeed contained less NR protein than Col-0 plants, and that decrease also corresponded with decreased NR activity (Figure 3B). Nevertheless, we also confirmed that, similar to that detected in roots (Figure 2B), the NO content in cotyledons of *prt6-1nlp7-1* plants was also significantly higher than in parental and wild-type plants (Figure 3C), even though the levels of NR protein and activity were severely diminished. These data suggest that in *prt6-1nlp7-1* plants, the NR-mediated production of NO is likely not the main source of NO. We checked whether changes in the levels of NR protein and activity might be the results of altered gene expression. We found that the expression of *NIA2* gene was slightly altered and neither *NIA1* nor *NLP7* gene expression was significantly altered upon treatment with the proteasome inhibitor MG132, NO, or both together (Supplemental Figure S1A). Besides, *NLP7* gene expression was not significantly altered and *NIA1* very little was affected by these treatments in wild-type, *prt6-1nlp7-1*, or the parental single mutant plants (Supplemental Figure S1B), thus suggesting the changes detected in NR protein and activity are likely due to altered stability of the proteins.

On the other hand, the high endogenous NO content in these plants may be responsible for the reduced growth phenotype observed in *prt6-1nlp7-1* plants. Supplemental Figure S2A shows that *prt6-1nlp7-1* plants grew slower than wild-type or their parental single mutant genotypes when grown in soil under long-day photoperiodic conditions. Similarly, arrested skotomorphogenic growth was also observed in etiolated *prt6-1nlp7-1* seedlings, which elongated less than parental *nlp7-1*, and that were already significantly shorter than wild-type hypocotyls (Supplemental Figure S2B). Expression of *NLP7* under its endogenous promoter fully complemented the short hypocotyl phenotype of *nlp7-1 and prt6-1nlp7-1* (Supplemental Figure S2B).

### Hypersensitivity to high sucrose, ABA, and submergence-induced hypoxia of *prt6nlp7* plants

**Figure 4:**
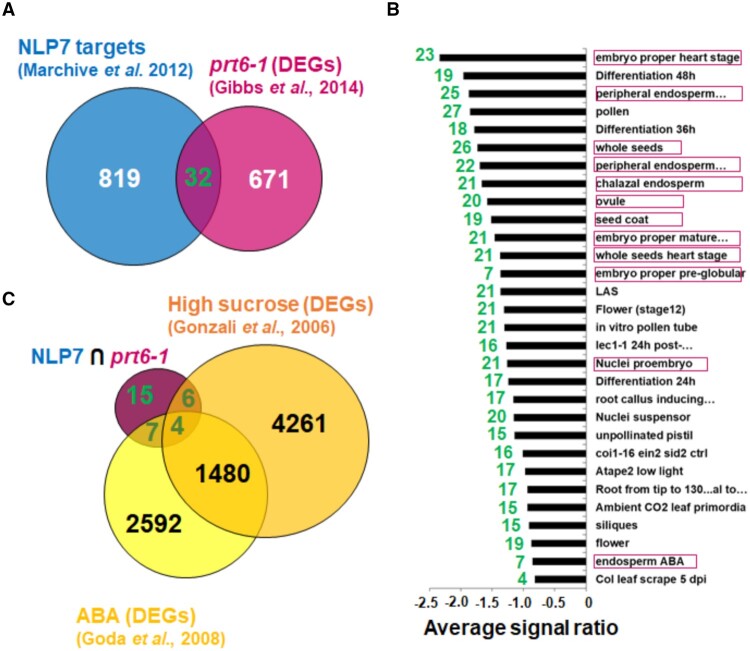
Overlap between NLP7 targets and DEGs related to PRT6, ABA, and sucrose. A, Venn diagram showing NLP7-target encoding DEGs in the *prt6-1* mutant. B, Gene Ontology analysis with AtCAST3.1 tools of genes in the intersection of (A) display enrichment of functional categories overrepresented among downregulated genes. Seed- and ABA-related categories are framed in red. C, Intersection between the 32 genes identified in (A) and DEGs identified in transcriptome analyses of plants treated with 10-µM ABA or 90-mM sucrose. The corresponding reference is shown below each group label.

**Figure 5:**
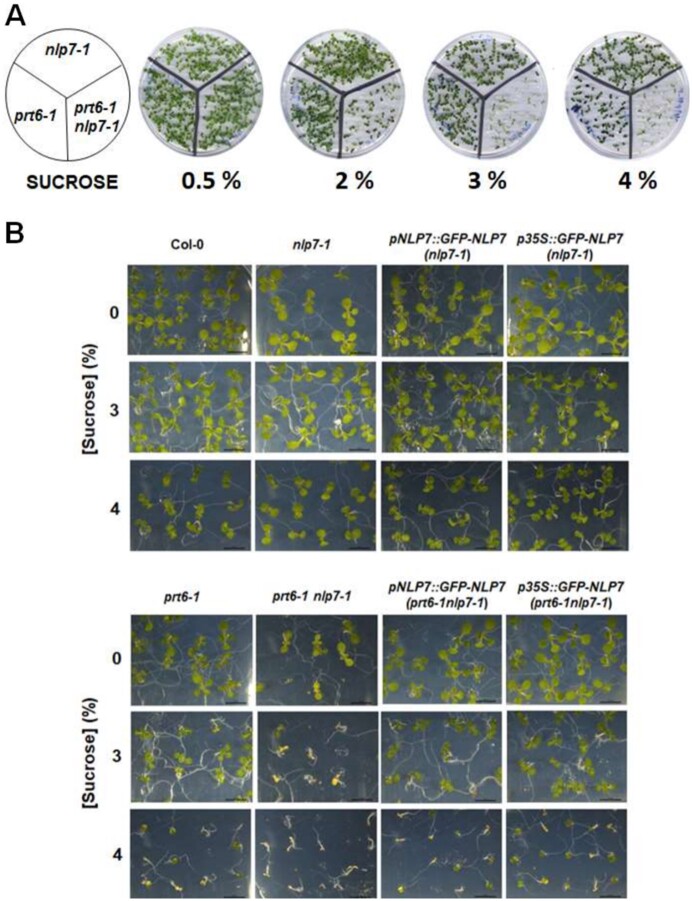
Seed germination and seedling establishment under high-sucrose concentrations. A, Appearance of seedlings from the indicated wild-type and mutant genotypes 7 d after sowing seeds in Murashige-Skoog (MS) medium containing the increasing sucrose concentrations indicated below the panels. B, Close-up, detailed view of seedlings of the indicated genotypes 5 d after sowing seeds in 0.5 × MS medium containing 3% and 4% (w/v) sucrose. The scale bars represent 1 cm.

To define what factors are involved in the functional interaction between NLP7 and PRT6, we searched for NLP7 targets (Marchive et al., 2013) that were differentially expressed in *prt6-1* mutant plants (Gibbs et al., 2014). Figure 4A shows that 32 genes were targeted by NLP7 and differentially expressed (DEGs) in *prt6-1* plants (Supplemental Table S1). A Gene Ontology analysis with those 32 DEGs showed an enrichment of seed-related categories (Figure 4B). Moreover, as shown in Figure 4C, around one-third of those genes were identified as DEGs based on the transcriptome analysis of plants treated with 90 mM sucrose (Gonzali et al., 2006) or 10 µM ABA (Goda et al., 2008), thus suggesting the functional link between NLP7 and PRT6 might be connected to seed-related responses/sensitivity to sucrose and/or ABA. It has been previously reported that *prt6* mutant seeds germinated poorly in medium supplemented with a high concentration of sucrose or ABA (Holman et al., 2009). We tested whether *prt6-1nlp7-1* seeds showed altered responses to high-sucrose concentrations. Figure 5 shows that *prt6-1nlp7-1* seeds were more sensitive than *prt6-1* seeds to sucrose. The enhanced sensitivity of *prt6-1* seeds required a sucrose concentration >3%, while *prt6-1nlp7-1* seeds showed hypersensitivity at sucrose concentrations of 2%, even though their parental *nlp7-1* seeds were fully insensitive (Figure 5A). In medium supplemented with 3% or 4% sucrose, wild-type Col-0 and *nlp7-1* seeds germinated and developed green cotyledons, *prt6-1* seedlings were partially growth arrested, and *prt6-1nlp7-1* seeds either germinated and developed chlorotic seedlings at 3% sucrose or were fully arrested in expanding cotyledons and severely chlorotic at 4% sucrose (Figure 5B). To test whether the hypersensitivity to high-sucrose concentration was due to the loss of *NLP7* expression, *pNLP7::GFP*–*NLP7 and 35S::GFP*–*NLP7* transgenic lines in *nlp7-1 and prt6-1nlp7-1* mutant genetic backgrounds were also tested for high-sucrose inhibition of seed germination. Figure 5B shows that *NLP7* overexpression and, to a lesser extent, the expression under its endogenous promoter, fully or partially complemented the sensitive phenotype of *prt6-1nlp7-1* seedlings at 3% or 4% sucrose, respectively (Figure 5A). The high-sucrose phenotype of seed germination was modulated by the N source used by the plant. Supplemental Figure S3 shows that NO_3_ attenuated the inhibition of seed germination and seedling establishment triggered by sucrose in all tested genotypes except *prt6-1nlp7-1*, which remained fully inhibited in all N-related test conditions. We have also tested *prt6-5*, another *prt6* mutant allele, in combination with *nlp7-1* and found that the high sucrose-triggered inhibition of seed germination was slightly lower with this mutant allele (Supplemental Figure S3). We also found that *NLP7* overexpression on the *prt6-1nlp7-1* background complemented the NLP7 loss of function to levels of sensitivity detected in *prt6-1* plants (Supplemental Figure S3).

**Figure 6:**
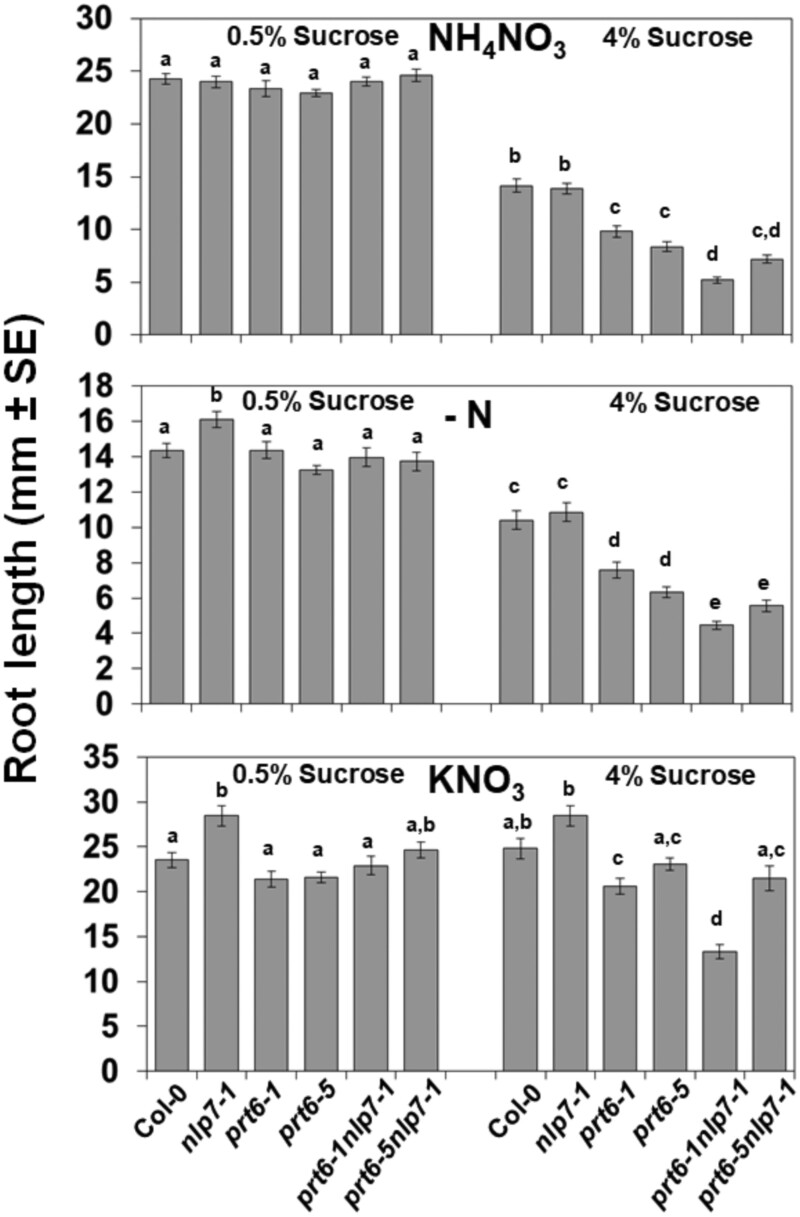
Sensitivity of primary root elongation to high sucrose. Primary root elongation of seedlings of the indicated genotypes grown vertically in plates with MS medium containing NO_3_ and NH_4_ (NH_4_NO_3_), MS medium without an N source (−N), or with 5 mM NO_3_ (KNO_3_) as the N source and the indicated sucrose concentration. The values are the mean of 25–50 plant roots ± se per genotype and condition. Statistical significance was calculated by one-way ANOVA test followed by Tukey’s HSD test for multiple comparisons. The letters indicate significant differences (*P* < 0.05).

The hypersensitivity of the *prt6-1nlp7-1* genotype to high sucrose was not restricted to seed germination. The elongation of the primary root was also significantly shortened in *prt6-1nlp7-1* seedlings compared with wild-type Col-0 or *nlp7-1* when grown in the presence of 4% sucrose but not in 0.5% sucrose (Figure 6). Roots of *prt6-1nlp7-1* seedlings were also shorter than *prt6-1* roots, which were already more sensitive to sucrose than wild-type roots (Figure 6). As mentioned above with the seed germination phenotype (Supplemental Figure S3), the high-sucrose inhibition of root elongation was also weaker with the *prt6-5nlp7-1* allele (Figure 6). The hypersensitive phenotype in *prt6-1nlp7-1* roots was observed independently of the N status of the plants, as was detected in medium without N and in medium containing potassium NO_3_ (KNO_3_) or NH_4_NO_3_ as N sources (Figure 6). However, the hypersensitive phenotype of *prt6-5 and prt6-5nlp7-1* roots to sucrose was largely attenuated with NO_3_ as the only N source (Figure 6). Although *prt6-1 and prt6-5* mutant alleles both have T-DNA insertions located very close in the third exon and they have been used interchangeably with equivalent qualitative phenotypes (Garzón et al., 2007; Holman et al., 2009; Gibbs et al., 2011, 2014), we found that *prt6-5* was less sensitive to high sucrose than *prt6-1*. This phenotype was somehow dependent on the N source, perhaps indicative of underlying functional interactions between carbon and N metabolism and signaling.

**Figure 7:**
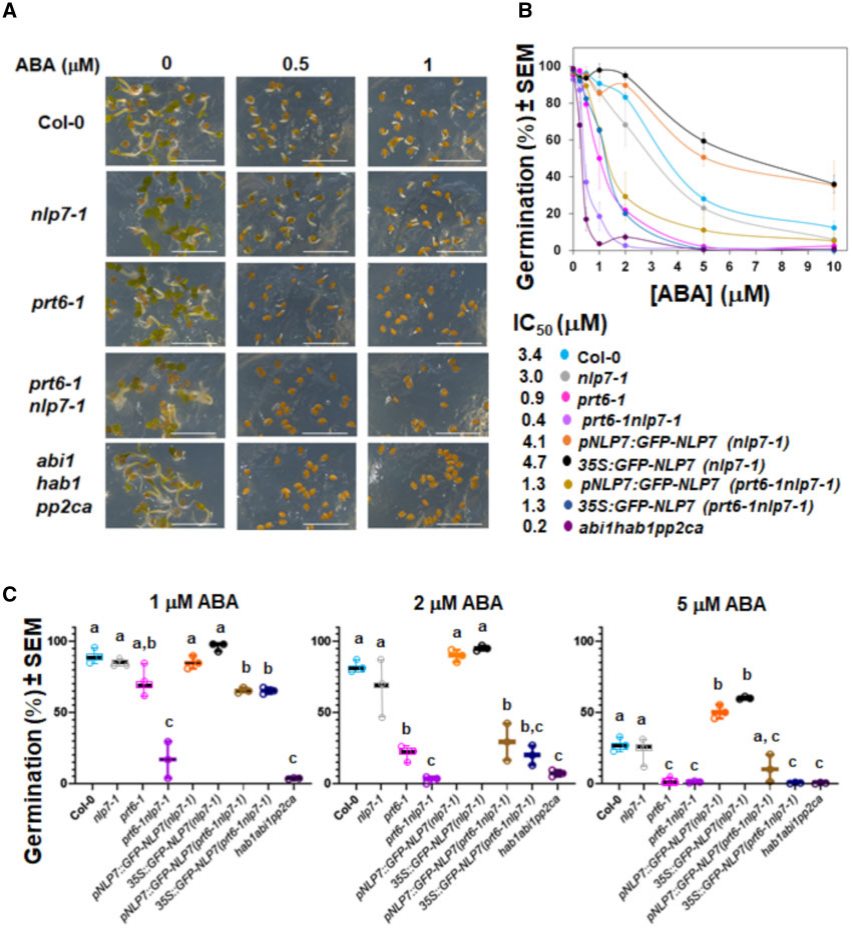
Sensitivity to ABA in the seed germination assays. A and B, Images of seed gemination assays and rates of seed germination, respectively, of the indicated mutant genotypes 72 and 48 h, respectively, after sowing seeds in 0.8% agar supplemented with the indicated ABA concentration. The values are the mean of three independent experiments ±se with around 25 seeds per genotype and condition. The half maximal inhibitory concentration values were estimated from the interpolation of data from curves. The scale bars represent 4 mm. C, Box plot graphs showing the individual points, the median line, and the max and min of the distribution, and one-way ANOVA followed by Tukey’s HSD statistical analysis of germination rates of the different genotypes at 1, 2, and 5 µM ABA. The letters indicate significant differences (*P* < 0.05).

As mentioned above, altered sensitivity to ABA might also be the basis of the NLP7–PRT6 functional interaction (Figure 4C). Seed germination assays with the same-age seeds for the above-mentioned NLP7-related mutant and transgenic genotypes harvested together were performed in medium supplemented with increasing concentrations of ABA. The data indicated that *prt6-1nlp7-1* seeds were strongly hypersensitive to ABA (Figure 7). The ABA-triggered inhibition of seed germination was similar in Col-0 and *nlp7-1* seeds, more intense in *prt6-1* mutant seeds as reported previously (Holman et al., 2009; Zhang et al., 2018), and extreme in *prt6-1nlp7-1* mutant seeds (Figure 7A). By 48 h after sowing seeds in 1 µM ABA, between 90% and 100% of the wild-type and *nlp7-1* seeds, around 50% of the *prt6-1* seeds, and ˂20% of the *prt6-1nlp7-1* seeds had germinated (Figure 7B). The hypersensitivity of *prt6-1nlp7-1* mutant seeds was comparable to that displayed by the triple *abi1-2hab1-1pp2ca-1* phosphatase 2C mutant (Figure 7, A and B), which has been characterized for its extreme response to exogenous ABA (Rubio et al., 2009). Hypersensitivity to ABA in germination paralleled a strong failure in establishing *prt6-1nlp7-1* seedlings even at lower tested ABA concentrations. No *prt6-1nlp7-1* seedling was established at 0.5 µM ABA, and *NLP7* expression on this background could not complement the arrested establishment phenotype (Supplemental Figure S4). However, the ABA-hypersensitive germination phenotype of *prt6-1nlp7-1* seeds was fully complemented to the levels of sensitivity of *prt6-1* seeds by the expression of *NLP7* (Figure 7B). Because *nlp7-1* did not display significantly different seed germination and seedling establishment rates than Col-0, the regulatory effects exerted by NLP7 on both processes should be linked to PRT6. However, independent regulatory actions exerted by NLP7 and PRT6 cannot be ruled out in controlling seedling establishment.

**Figure 8:**
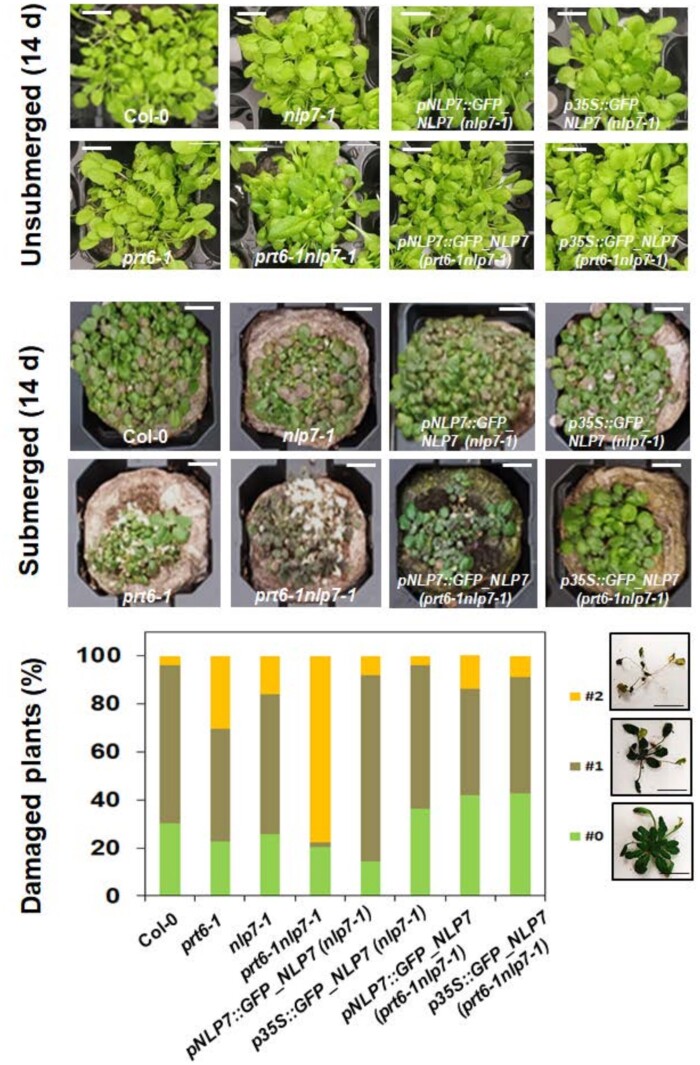
Tolerance to submergence-triggered hypoxia. Seedlings of the indicated genotypes were submerged under dim light or unsubmerged as a control for 5 d. After submergence, plants were re-oxygenated for 14 d. Representative images of seedlings of the different genotypes after recovery or the control unsubmerged are shown in the top panels as indicated. The bottom panel shows the quantification of plant damage 14 d after re-oxygenation recovery. The degree of damage was calculated according to a three-stage classification as indicated at the right seed (0, not damaged, in green; 1, damaged, in brown; 3, dead, in yellow). The scale bars represent 1 cm.

In addition to the high sucrose and ABA-hypersensitive phenotypes, we also checked whether *prt6nlp7* plants might be affected in another PRT6-related phenotype, the tolerance to submergence-induced hypoxia followed by reoxygenation recovery. For that, Col-0, *nlp7*, *prt6*, *prt6nlp7*, and NLP7-overexpressing plants on those backgrounds were submerged for 5 d under dim light and then reoxygenated back by removing water and allowing for recovery. After 14 d of reoxygenation recovery, the plant survival rate for each genotype was scored by classifying individuals as nondamaged, partially damaged, or dead. Single *nlp7-1 and prt6-1* plants were already less tolerant to submergence than Col-0 plants (Figure 8). These data on *prt6-1* tolerance to submergence are consistent with some previous reports (Licausi et al., 2011; Weits et al., 2014) but are contradictory with other studies (Gibbs et al., 2011; Riber et al., 2015), thus highlighting the importance of factors such as light conditions and humidity during recovery for determining plant survival (Riber et al., 2015). The phenotype was potentiated in the *prt6nlp7* plants and fully or partially complemented by *NLP7* expression under its endogenous or *35S* promoter, respectively (Figure 8).

### Could NLP7 be a substrate of the PRT6-mediated N-degron proteolytic pathway?

**Figure 9:**
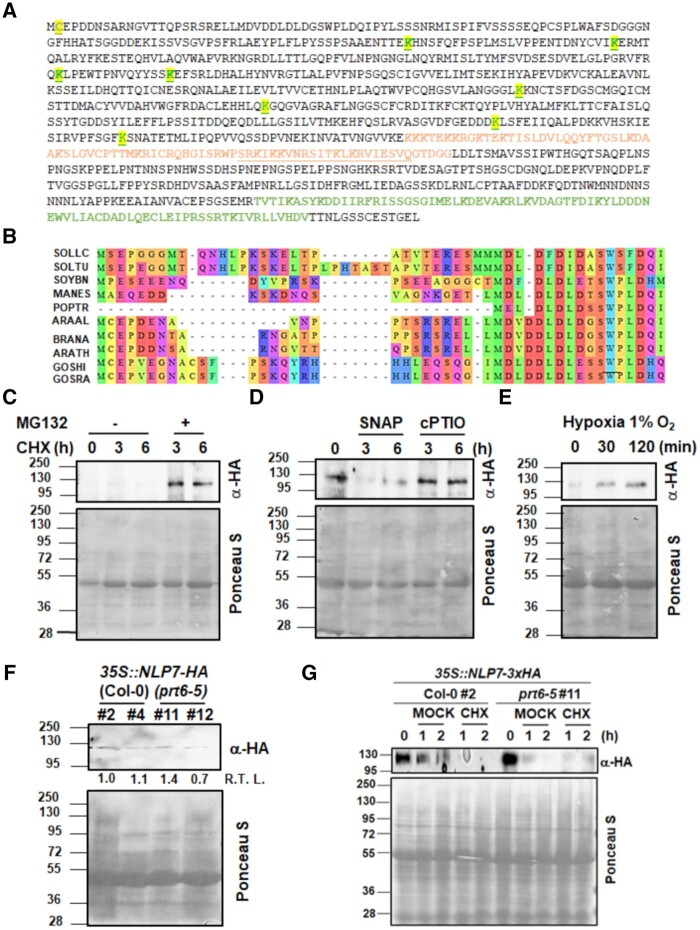
NLP7 protein polyubiquitination and degradation. A, Amino acid sequence of NLP7 protein showing the RWP-RK (orange) and PB1 (green) domains, the Cys2 in blue, and the Lys (K) residues predicted to be ubiquitinated in green and underlined. B, Alignment of the N-terminus of NLP7 putative orthologs in different plants. SOLLC, *Solanum licopersicum*; SOLTU, *Solanum tuberosum*; SOYBN, *Glycine max*; MANES, *Manihot esculenta*; POPTR, *Populus tremula*; ARAAL, *Arabis alpina*; BRANA, *Brassica napus*; ARATH, *A. thaliana*; GOSHI, *Gossypium hirsutum*; GOSRA, *Gossypium raimondii*. C–E, NLP7-HA protein levels were analyzed by western blot with anti-HA antibodies (α-HA) in untreated (−) plants or treated (+) with cycloheximide (CHX), proteasome inhibitor (MG132), NO scavenger (cPTIO), NO donor (SNAP), or hypoxia for the indicated times, respectively. F, Levels of NLP7-HA protein in transgenic overexpressing lines in Col-0 and *prt6-5* backgrounds. Relative transgene levels are shown below the top panel for each line. G, Levels of NLP7-HA protein in transgenic overexpressing lines on the Col-0 and *prt6-5* backgrounds were analyzed at the indicated times either in 100 µM CHX-treated or untreated control (MOCK) plants. Exposure times during image acquisition were optimized to better display differences between treatments. The positions of the molecular mass markers (kDa) are shown to the left side of the western blot and Ponceau S-stained gel, used as a loading control.

PRT6 catalyzes the polyubiquitination of proteins through the Cys/Arg N-degron pathway that removes the starting Met, then oxidizes the exposed Cys residue and further arginylates it before being ubiquitinated (Garzón et al., 2007; Gibbs et al., 2011). For NLP7 to be a PRT6 substrate, NLP7 should have a Cys residue in position 2 and should also be potentially ubiquitinated. Indeed, NLP7 has a Cys2 (Figure 9A). Besides, an in silico analysis pointed to NLP7 being potentially ubiquitinated at different Lys residues (Figure 9A;Supplemental Table S2). However, the fact that the Cys2 residue is conserved only in some NLP7 orthologs, including those from Brassicas and Gossypium but not those from the family Solanaceae or the genus Populus (Figure 9B), raises some doubts about the potential physiological relevance of an N-degron pathway-based regulatory mechanism on NLP7 action. We have generated transgenic plants overexpressing C-terminal hemagglutinin (HA)-tagged NLP7, thus preserving its potential N-terminal degron sequence, on both the Col-0 and *prt6-5* backgrounds. We found that NLP7-HA protein was stabilized in transgenic plants on the Col-0 background by several treatments including the proteasome inhibitor MG132 under *de novo* protein synthesis inhibition with cycloheximide (Figure 9C), the NO scavenger 2-(4-carboxyphenyl)-4,4,5,5-tetramethylimidazoline-1-oxyl 3-oxide (cPTIO; Figure 9D), or hypoxic conditions in 1% O_2_ (Figure 9E). In turn, treatment with the NO donor S-nitroso-N-acetylpenicillamine (SNAP) led to protein degradation (Figure 9D). These are typical features of proteins characterized as substrates of the PRT6 branch of the N-degron pathway (Gibbs et al., 2011, 2018; Weits et al., 2019). However, when comparing the basal levels of NLP7-HA protein in the independent *35S::NLP7-HA* lines on the Col-0 and *prt6-5* mutant background, we found that plants from lines with similar levels of transgene expression for both backgrounds also showed similar levels of protein (Figure 9F). Moreover, treatment with the protein synthesis inhibitor CHX led to full protein degradation in *35S::NLP7-HA* plants both on both the Col-0 and *prt6-5* backgrounds (Figure 9G), thus suggesting PRT6-triggered ubiquitination was not required for further NLP7 degradation. Partial NLP7 protein degradation was also observed in the corresponding mock-treated samples (Figure 9G), likely reflecting a circadian clock-controlled NLP7 protein levels.

**Figure 10:**
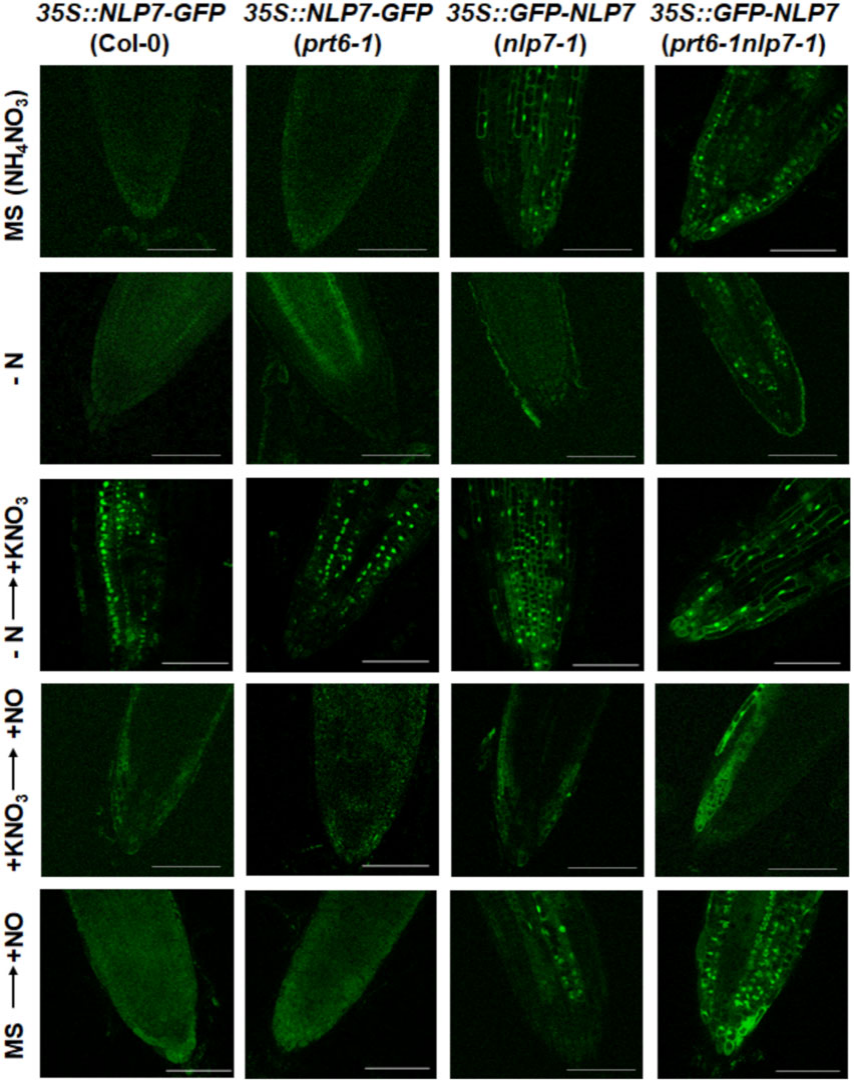
Effect of NO_3_ and NO on GFP-tagged NLP7 protein. GFP was visualized by confocal microscopy in root tips of plants of the indicated genotypes after transference from MS medium with NH_4_NO_3_ as the N source to MS medium without N source (−N) for 3 d, then supplemented with 5 mM KNO_3_ (−N → +KNO_3_) for 2 h. Both NO_3_-induced and NH_4_NO_3_ grown plants were exposed to a pulse of 300 ppm NO for 5 min and images were taken after 2 h (+KNO_3_ → +NO and MS → +NO, respectively). The scale bars represent 100 µm.

It had been reported that NLP7 is translocated to and retained in the nucleus in response to NO_3_ (Marchive et al., 2013). We have generated transgenic lines overexpressing C-terminal GFP-tagged NLP7 on the Col-0 and *prt6-1* backgrounds as well as N-terminal GFP-tagged NLP7 on the *nlp7-1 and prt6-1nlp7-1* backgrounds. N-terminal tags shield the potential N-degron sequence of NLP7, thus ruling out potential proteolysis through the Cys/Arg N-degron pathway. In turn, C-terminal tags do not interfere with that regulatory pathway. In standard Murashige–Skoog (MS) medium containing NH4NO3as the N source, only plants overexpressing GFP–NLP7 on the *nlp7-1 and prt6-1nlp7-1* backgrounds displayed nuclear fluorescence that disappeared or was severely reduced after transference of plants to medium without any N source (Figure 10). We confirmed that by transferring plants of each genotype from medium without N to medium supplemented with 5 mM NO_3_ for 2 h, all plants showed fluorescence in the nuclei (Figure 10). Interestingly, fluorescence disappeared from nuclei in *35S::NLP7*–*GFP* plants on both the Col-0 and *prt6-1* backgrounds as well as in *35S::GFP*–*NLP7*(*nlp7-1*) plants after NO treatment of NO_3_-induced plants, and it was substantially reduced in *35S::GFP*–*NLP7*(*prt6-1nlp7-1*) plants (Figure 10). A similar NO treatment of plants grown continuously in MS with NH_4_NO_3_ led to a reduction in nuclear fluorescence for plants on the *nlp7-1* background while the nuclear fluorescence remained in plants on the *prt6-1nlp7-1* background (Figure 10). These data suggest that PRT6 might be involved in NO-triggered degradation and/or nuclear export of NLP7. The fact that these transgenic plants expressed a GFP–NLP7 fusion protein, thus shielding the potential N-degron sequence, indicated that the PRT6 action was not exerted on NLP7 as a potential substrate of the PRT6-mediated N-degron proteolysis, but rather through a still unknown PRT6-regulated protease or component of the nuclear export machinery. In agreement, the transference from N starvation to medium supplemented with NO_3_ led to similar fluorescence in the nuclei for wild-type and *prt6-1* backgrounds, thus indicating the loss of PRT6 function did not entail increased stability of the NLP7–GFP protein, again suggesting NLP7 is not a PRT6-mediated N-degron pathway substrate. However, fluorescence was excluded from nuclei and detected in the cytoplasm upon treatment with NO in transgenic plants expressing C-terminal GFP-tagged NLP7 protein on both the Col-0 and *prt6-1* backgrounds (Figure 10). These data suggest PRT6 is not necessary to allow the NO-triggered exclusion of NLP7 from nuclei, and also that if the N-terminus sequence is involved in this process, it should be mediated by a recognin different from PRT6. Because nuclear localization of transgenic GFP–NLP7 seems to be maintained in NO-treated *prt6-1nlp7-1* plants (Figure 10), simultaneous and opposite potential roles of PRT6 and endogenous NLP7 in regulating the nuclear export of GFP–NLP7 might be relevant.

## Discussion

The transcription factor NLP7 plays a key regulatory role on NO_3_ assimilation by activating the gene expression of *NR* and others (Castaings et al., 2009; Konishi and Yanagisawa, 2013; Marchive et al., 2013; Yu et al., 2016; Cao et al., 2017; Zhao et al., 2018). Consequently, NLP7 function might directly affect the NR-mediated production of NO from nitrite. On the other hand, the E3 ubiquitin ligase PRT6 is a well-characterized recognin of the Cys2-containing N-degrons of ERFVII transcription factors that together are involved in NO sensing in Arabidopsis (Gibbs et al., 2014). Because NLP7, like the few PRT6 substrates characterized to date (Gibbs et al., 2011, 2018; Weits et al., 2019; Labandera et al., 2021), is a Cys2-containing protein, the possibility that NLP7 could be a PRT6 substrate is worth studying. That would represent a hub between N-degron proteolysis and NO_3_ signaling and assimilation, which would require better attention. Our findings in this work do not support the hypothesis that NLP7 is a PRT6 substrate. Although being stabilized by inhibition of the proteasome (Figure 9C), by scavenging NO (Figure 9D), or by hypoxia (Figure 9E), all features of PRT6 substrates (Gibbs et al., 2011, 2018; Weits et al., 2019), there was not enhanced stabilization was detected in plants expressing C-terminal HA-tagged (Figure 9F) or GFP-tagged (Figure 10) NLP7 on the *prt6* mutant background compared with the wild-type background. Therefore, NLP7 cannot be considered a PRT6 substrate, even though it cannot be ruled out that NLP7 could be regulated by the Cys/Arg branch of the N-degron pathway but as a substrate of a still unknown recognin. However, to our knowledge, no other recognin different from PRT6 has been identified as a component of that N-degron pathway. Alternatively, NLP7 might be indirectly regulated by a PRT6 substrate. We have analyzed the −1,000 bp promoter region of *NLP7* locus with AthaMap (www.athamap.de/; Hehl et al., 2016) searching for potential binding sites of transcription factors already characterized as PRT6 substrates. Among them, we found the TGCAGCCGTC motif in the *NLP7* promoter sequence that contains a putative RAP2.3 (ERFVII) binding site (Franco-Zorrilla et al., 2014) located 388-bp upstream of the start codon. Thus, a RAP2.3-mediated regulation of NLP7 by PRT6 cannot be thus ruled out. Regarding this possibility, we have recently reported that RAP2.3 seemed to work as a molecular rheostat controlling NO homeostasis and signaling (León et al., 2020), but our transcriptome data of transgenic plants conditionally expressing *RAP2.3* or *RAP2.12* do not support the hypothesis of *NLP7* being a target of ERFVII transcriptional regulation.

Although we detected NLP7-HA protein accumulation and degradation in plants treated with the NO scavenger cPTIO and the NO donor SNAP, respectively (Figure 9D), treatment of *35S::NLP7*–*GFP* plants with exogenous NO gas did not lead to fluorescence reduction, but instead to nucleocytoplasmic shuttling (Figure 10). In silico prediction of a potential nuclear export signal (NES) in the NLP7 protein pointed to the existence of a likely functional NES (Supplemental Figure S5). The motif SRSRELLMDVDDLDL (Supplemental Figure S5B)—or the more restricted motif MDVDDLDL (Supplemental Figure S5C)—in the N-terminus was predicted as an NES with a high score. It has been widely reported that in mammals the SUMOylation of certain proteins controls their nucleocytoplasmic transit (Ptak and Wozniak, 2017). NLP7 is predicted to be potentially SUMOylated with high score for Lys845 and Lys889 (Supplemental Table S2; Supplemental Figure S6B), the latter being located inside the PB1 domain of the protein (Supplemental Figure S6A). In agreement with the predicted SUMOylation of NLP7, western blot analysis with plants overexpressing NLP7-3xHA showed a band close to 130 kDa (Figure 9) that is larger than the theoretical 115 kDa expected for the unmodified tagged protein. ABA hypersensitivity in seed germination and seedling root growth inhibition in the *siz1* mutant was due to the reduced SUMOylation of the ABI5 transcription factor (Miura et al., 2009). The nucleocytoplasmic shuttling of Exportin 1-interacting WD40 protein 1 controls the stability of ABI5 and additional ABA-triggered responses (Xu et al., 2019). Another factor translocated to the nucleus, FYVE DOMAIN PROTEIN REQUIRED FOR ENDOSOMAL SORTING 1, transcriptionally inhibited ABA signaling (Li et al., 2019) and mediated a dynamic turnover of ABA receptors from the plasma membrane to the endosomal/vacuolar degradation pathway (Belda-Palazon et al., 2016). These data support a potential link between NLP7 function and ABA-regulated processes through SUMOylation-controlled transport between the cytoplasm and the nucleus. Interestingly, NLP7 is predicted to be N-glycosylated at several Asn residues (Supplemental Figure S6C). N-glycosylation has been widely characterized as a PTM involved in the control of protein folding and quality control in the endoplasmic reticulum (Nagashima et al., 2018). This modification may represent another functional link between NLP7 and vesicle-trafficking-related events involved in ABA signaling. Whether the potential SUMOylation of NLP7 and nucleocytoplasmic shuttling may have physiological relevance in determining the hypersensitivity of the *prt6-1nlp7-1* mutant in the ABA-related phenotypes described in this work needs to be further studied.

We presented data supporting that NLP7 is required for NR expression (Figure 3, A and B) and that NR protein and activity were enhanced upon treatment with proteasome inhibitors both in NH_4_- and NO_3_-grown plants (Figure 1, B and C). We previously reported that Arabidopsis NIA1 and NIA2 are ubiquitinated (Costa-Broseta et al., 2021), thus supporting that NR levels are regulated through polyubiquitination and subsequent proteasomal degradation. NRs are also SUMOylated and activated by SIZ1 (Park et al., 2011), and it has been proposed that the NR–SIZ1 interaction could help to relocalize NRs to the nucleus in a process that is repressed by NH_4_ (Kim et al., 2018). On the other hand, NRs are also phosphorylated and then become inactivated (Lambeck et al., 2012), thus suggesting SUMOylation and phosphorylation have antagonistic effects on NRs. The fact that NLP7 is also phosphorylated (Liu et al., 2017) and potentially SUMOylated (Supplemental Figure S6B) as well as located both in the cytoplasm and nucleus represents an interesting parallelism suggesting that the posttranslational status of NLP7 and NRs largely determine their function and fate.

PTMs linked to NO action were also predicted to occur in NLP7, including the nitration of Tyr157 and Tyr288 as well as the S-nitrosation of Cys2 and Cys374 (Supplemental Figure S6C). These PTMs were also identified in NRs (Costa-Broseta et al., 2021), again suggesting PTMs are relevant for the regulation of the NLP7-NR-regulated processes. Among them, the production of NO—which was reduced in the *nlp7-1* (Figures 2, B and 3, C) and *nia1nia2* (Lozano-Juste and León, 2010) mutants, and enhanced upon overexpression of *NRs* (Costa-Broseta et al., 2021) and *NLP7* (Figure 2B)—is unexpectedly potentiated in *prt6-1nlp7-1* plants (Figures 2B and 3C). The enhanced production of NO under conditions with strongly reduced NR function point to the existence of NR-independent NO biosynthetic mechanisms that could be negatively regulated by PRT6 and/or NLP7, but future work will be necessary to address this eventually. We have described here several other phenotypes such as sensitivity to high sucrose (Figures 5 and 6) or ABA (Figure 7) as well as tolerance to submergence-triggered hypoxia (Figure 8), in which the loss of NLP7 function potentiated the phenotypes of the *prt6-1* mutant plants in a similar manner to that described for NO production. Taken together, these findings suggest that there are multiple functional links among NLP7, NRs, and PRT6 that connect NO_3_ assimilation and signaling with the PRT6 branch of the N-degron pathway, and likely with ABA signaling.

## Materials and methods

### Plant materials

Arabidopsis (*A.* *thaliana*) seeds were surface sterilized with chlorine gas before sowing in MS-MES medium plates containing 1% (w/v) sucrose. Seeds from Col-0 and *nlp7-1* mutant (SALK_026134) were obtained from Nottingham Arabidopsis Stock Center. Seeds from *prt6-1 and prt6-5* were obtained from Michael Holdsworth (University of Nottingham, UK) and *abi1-2hab1-1pp2ca-1* from Pedro Rodriguez (IBMCP, Valencia, Spain). Seeds expressing N-terminal GFP-tagged NLP7 under its endogenous or *35S* promoter in *nlp7-1* mutant background were obtained from Anne Krapp (INRAE-Institut Jean Pierre Bourgin, France). Plants overexpressing C-terminal 3×HA- or GFP-tagged versions of NLP7 were generated by Gateway subcloning of the full-length cDNAs (obtained by PCR with NLP7-F and nostopNLP7-R primers described in Supplemental Table S3) in *pGWB14 and pGWB5* vectors, respectively, and further transformation of *Agrobacterium tumefaciens* C58 with the corresponding constructs. Col-0, *prt6-1* or *prt6-5* plants were then genetically transformed by dipping floral organs in a suspension of transformed *A. tumefaciens* (Clough and Bent, 1998) and selected for homozygotic transgenes by screening kanamycin or hygromycin resistance depending on the vectors used. The double *prt6-1nlp7-1* and *prt6-5nlp7-1* plants were obtained by crossing the corresponding single mutants and the subsequent identification of homozygous individuals by PCR-assisted genotype with specific primers (Supplemental Table S3). *pNLP7::GFP*–*NLP7(prt6-1nlp7-1) and 35S::GFP*–*NLP7(prt6-1nlp7-1)* plants were generated by crossing the corresponding transgenic lines on the *nlp7-1* background mentioned above with *prt6-1nlp7-1* plants and subsequent PCR-assisted genotyping with specific primers for *prt6-1* T-DNA insertion (Supplemental Table S3).

### Growth conditions and treatments

Arabidopsis seeds were sown in either complete MS medium (Duchefa Biochemie, The Netherlands) containing NH_4_NO_3_ as the N source, or in modified MS medium without N (bioWORLD, Irving, TX, USA) and then supplemented with 5 mM KNO_3_, 5 mM NaNO_2_ or 2.5 mM (NH_4_)_2_SO_4_ as the N source as indicated. NO treatments were performed by exposing plants to a pulse of 300 ppm of pure NO gas (Linde AG, Germany) during 5 min in a tightly sealed transparent box. Scavenging of NO was performed by treatment with 0.2 mM cPTIO (Sigma, Burlington, MA, USA). As a NO donor, treatment with 0.2 mM SNAP (Merck, Darmstadt, Germany) was performed. Inhibition of protein synthesis and proteasome-mediated degradation was performed by treating plants with 50 µM CHX and 100 µM carbobenzoxy-Leu-Leu-leucinal (MG-132), respectively (Sigma, USA).

Hypocotyl length was measured for every seedling of each genotype and condition tested by using Image J2/Fiji. Hypocotyl assays were performed with etiolated seedlings grown for 4 d under darkness. The experiments were repeated three times with at least 20 individuals per genotype, condition, and experiment. To test the effect of sucrose on seed germination and primary root elongation, seeds of the same age of the different genotypes were sown in 0.5 × MS plates containing 0.8% (w/v) agar supplemented with 1%–4% (w/v) sucrose and photographed after 5 and 7 d for the germination and root assays, respectively. The root length was calculated by using Image J2/Fiji. The analyses of seed germination rates were performed by sowing seeds in 0.8% (w/v) agar supplemented with ABA at different concentrations between 0.5 and 10 µM. After 3 d of stratification at 4°C in darkness, plates were incubated under photoperiodic conditions (16-h light: 8-h darkness), and the germination rates were calculated at 48 h after exposure to light. The rates were calculated as the mean of three independent experiments with around 150 seeds per genotype and condition.

Experiments under hypoxic conditions were performed either by submergence or by incubation in a box containing an inlet for N_2_ gas and an O_2_ sensor connected to a ProOx Model 110 controller that allows a tight control of the O_2_ concentration inside the box (BioSpherix, Parish, NY, USA). Submergence experiments were performed with plants sown in Jiffys (Jiffy Products International AS, Norway) and grown under a short-day-photoperiod (8-h light: 16-h darkness) for 3 weeks. Plants were arranged in a randomized complete block design with two replicates per genotype and 20–30 seedlings per Jiffy. Plants were submerged under 20-cm-deep water and kept under dim light to simulate the conditions in deep floodwater for 5 d. The photon flux density reaching the plants under the shades was ˂2 μmol m^−2^ s^−1^. At the end of the submergence treatments, the water was removed, and plants were transferred to the standard long-day photoperiodic conditions at a photon flux density of 100 μmol m^−2^ s^−1^. Recovery was monitored for 14 d and the survival rate was calculated by using a three-stage classification: undamaged, partially damaged, or dead. The experiments with the O_2_-controlled chamber were performed with 14-d-old plants grown in vitro under the long-day photoperiodic conditions (100 μmol m^−2^ s^−1^) in MS medium supplemented with 0.5% (w/v) sucrose. Hypoxia treatment was performed under low light intensity (20 μmol m^−2^ s^−1^) at 1% (v/v) O_2_ for the indicated time, and the samples were collected right after finishing the hypoxia treatment and frozen in liquid N for protein extraction.

### Measurement of endogenous NO content and confocal microscopy

The endogenous levels of NO in shoots and roots were determined by staining with 10 µM 4-amino-5-methylamino-2',7'-difluorofluorescein diacetate (DAF-FM DA) fluorescein (Merck, Madrid, Spain) as described previously (Guo et al., 2003) with slight modifications. Fluorescence was detected by using a Zeiss (Oberkochen, Germany) LSM 780 confocal microscope (with excitation at 488 nm and emission at 500–527 nm range; bandwidth 489–550; gain 1,250) or with a Leica (Wetzlar, Germany) DM 5000B fluorescence microscope with a barrier filter to avoid chlorophyll autofluorescence, using unchanged parameters for every measurement. The specificity of NO-related fluorescence detection was assessed by treatment with 0.5 mM cPTIO or with 0.5 mM salicylic acid (SA) as an NO inducer. The DAF-FM DA fluorescence intensities were analyzed using Adobe Photoshop 7.0 by quantifying green pixels in three to six replicate images taken from independent plants in at least three different pots for every genotype and condition. The number of pixels was always normalized for the cotyledon or root area in each image.

The fluorescence of GFP-tagged NLP7 protein was visualized with a Zeiss LSM 780 confocal microscope (with excitation at 488 nm and emission at 500–527 nm range; bandwidth 489–550; gain 1,250) or a Leica DM 5000B fluorescence microscope in cotyledons or primary root tips, respectively.

### RNA isolation and reverse transcription quantitative PCR

RNA was extracted and purified with Nucleospin RNA Plant kit (Macherey-Nagel, Düren Germany), reverse transcribed with M-MuLV Reverse transcriptase (RNase H minus) and oligo-dT, and the resulting cDNA was quantified by reverse transcription quantitative PCR with an Applied Biosystems (Waltham, MA, USA) ABI 7500 Fast Real-Time Thermocycler by using specific primer pairs for *NIA1*, *NIA2*, and *NLP7*, with *ACT2* as a reference gene (Supplemental Table S3).

### Western blot analyses and NO_3_ reductase activity assay

The levels of NR and HA-tagged proteins were analyzed in total protein extracts by SDS–PAGE, blotting onto nitrocellulose membranes and further probing with polyclonal anti-NR (1:1,000 dilution; Agrisera, Sweden) and anti-HA-Horse radish peroxidase (1:1,000 dilution; Roche, Switzerland) antibodies. Loading control was assessed by staining nitrocellulose membranes after blotting with Ponceau S. NR activity assays were performed as reported previously (Park et al., 2011) with slight modifications (Costa-Broseta et al., 2021). Assays included 20 µg of protein extracts in a 250-µL total volume and were performed at 25°C for 30 min.

### In silico analyses and predictions

Amino acid sequences were aligned by using Clustal Omega (https://www.ebi.ac.uk/Tools/msa/clustalo/). Gene Ontology Consortium tools (http://www.geneontology.org) were used to analyze enrichment of functional categories; and AtCAST3.1 (http://atpbasmd.yokohama-cu.ac.jp/cgi/atcast/search_input.cgi) was used to compare publicly available transcriptome datasets. Prediction of nitration, S-nitrosation, and SUMOylation was performed by using tools from the Cuckoo Group (http://www.biocuckaoo.org/). N-glycosylation was predicted by using NetNGlyc version 1.0 (http://www.cbs.dtu.dk/services/NetNGlyc/).

### Statistical analyses

The values of transcript levels, NO levels, hypocotyl and root lengths, and seed germination rates are presented as the mean of at least three independent biological replicates ±se. Statistical significance was analyzed as indicated in the figure legends either by unpaired Student’s *t* test for the transcript levels in treated versus mock comparisons or one-way ANOVA test followed by Tukey’s honestly significant difference test for multiple comparisons between genotypes.

### Accession numbers

Arabidopsis accession numbers of the genes studied according to The Arabidopsis Information Resource are as follows: *NIA1*, At1g77760; *NIA2*, At1g37130; *NLP7*, At4g24020; *PRT6*, At5g02310/At5g02300.

## Supplemental data

The following supplemental materials are available in the online version of this article.

**Supplemental Figure S1.** Effect of the proteasome inhibitor MG132 and NO on *NIA1*, *NIA2*, and *NLP7* transcript levels.

**Supplemental Figure S2.** Vegetative growth phenotype of mutant and NLP7-overexpressing plants.

**Supplemental Figure S3.** Seedling establishment in high-sucrose concentration.

**Supplemental Figure S4.** Effect of ABA on seedling establishment.

**Supplemental Figure S5.** NES in the NLP7 protein.

**Supplemental Figure S6.** Predicted PTMs in the NLP7 protein.

**Supplemental Table S1.** Genes targeted by NLP7 and DEGs in *prt6-1* plants.

**Supplemental Table S2.** In silico analysis of Lys ubiquitylation for the NLP7 protein.

**Supplemental Table S3.** Oligonucleotides used in this work.

## Supplementary Material

kiab382_Supplementary_Data

## Notes

J.L. conceived the project; J.L. and M.C.C. designed the experiments; M.C.C., A.C.B., B.G., and J.L. carried out the experiments; J.L., M.C.C., and A.C.B. analyzed the data; J.L. wrote the article; and all authors approved the article for publication.

The author responsible for distribution of materials integral to the findings presented in this article in accordance with the policy described in the Instructions for Authors (https://academic.oup.com/plphys/pages/general-instructions) is: José León (jleon@ibmcp.upv.es).
