# Supplementary material for: NIN-like protein7 and PROTEOLYSIS6 functional interaction enhances tolerance to sucrose, ABA, and submergence
Source: Plant Physiol. 2021 Aug 10;187(4):2731–48. doi: 10.1093/plphys/kiab382 (PMC8644111; doi:10.1093/plphys/kiab382)
Supplement: kiab382_Supplementary_Data [file kiab382_supplementary_data.pdf]

Supplemental Data of Castillo et al.

The following supplemental materials are available.

Supplemental Figure S1. Effect of the proteasome inhibitor MG132 and nitric oxide (NO) on *NIA1*, *NIA2* and *NLP7* transcript levels.

Supplemental Figure S2. Vegetative growth phenotype of mutant and NLP7-overexpressing plants.

Supplemental Figure S3. Seedling establishment in high sucrose concentration.

Supplemental Figure S4. Effect of abscisic acid (ABA) on seedling establishment.

Supplemental Figure S5. Nuclear export signal (NES) in the NLP7 protein.

Supplemental Figure S6. Predicted post-translational modifications (PTMs) in the NLP7 protein.

Supplemental Table S1. Genes targeted by NLP7 and differentially expressed (DEGs) in *prt6-1* plants.

Supplemental Table S2. *In silico* analysis of Lys ubiquitylation for the NLP7 protein.

Supplemental Table S3. Oligonucleotides used in this work.

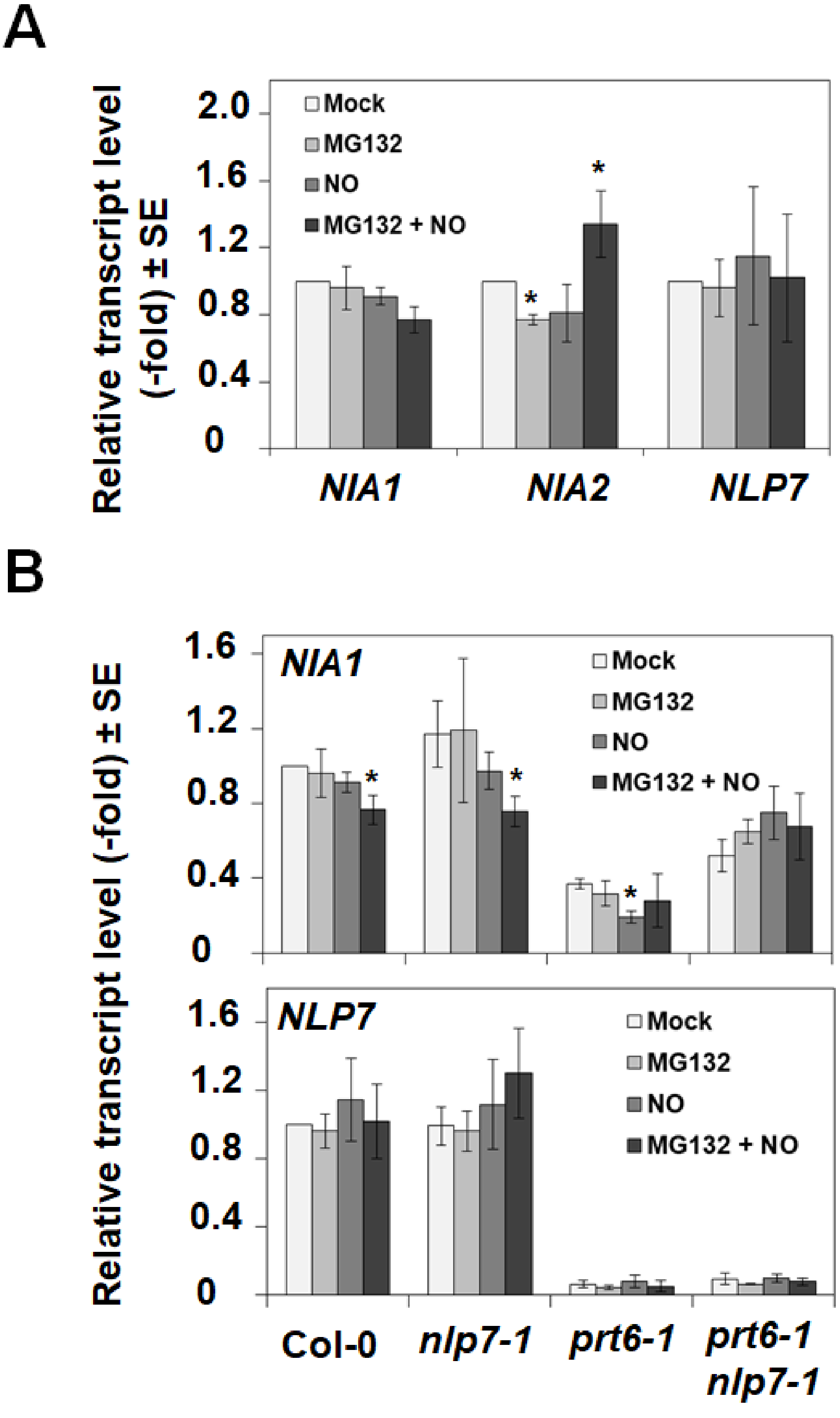

Supplemental Figure S1. Effect of the proteasome inhibitor MG132 and nitric oxide (NO) on *NIA1*, *NIA2* and *NLP7* transcript levels. A, Levels of the indicated gene transcripts in Col-0 plants either untreated (Mock) or treated with a pulse of 300 ppm NO during 5 min (NO), with 200 mM proteasome inhibitor (MG132) or both together (MG132+NO). The samples for total RNA extraction were collected 1h after treatments. B, *NIA1* and *NLP7* transcript levels in wild type Col-0 and the indicated mutant plants under the treatments described in panel A. The values of transcript levels are the mean of three independent biological replicates ± SE relatives to the levels of Mock treated Col-0 plants. Statistically different in unpaired Student´s t-test. (*) represents p-values <0.05 when compared with the corresponding mock treatment.

A

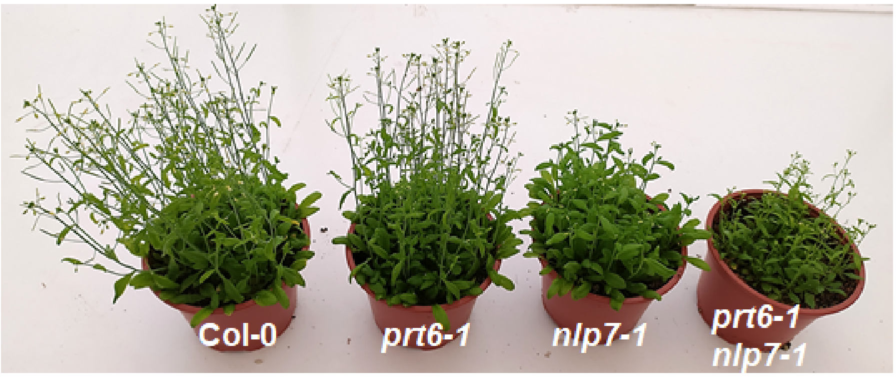

B

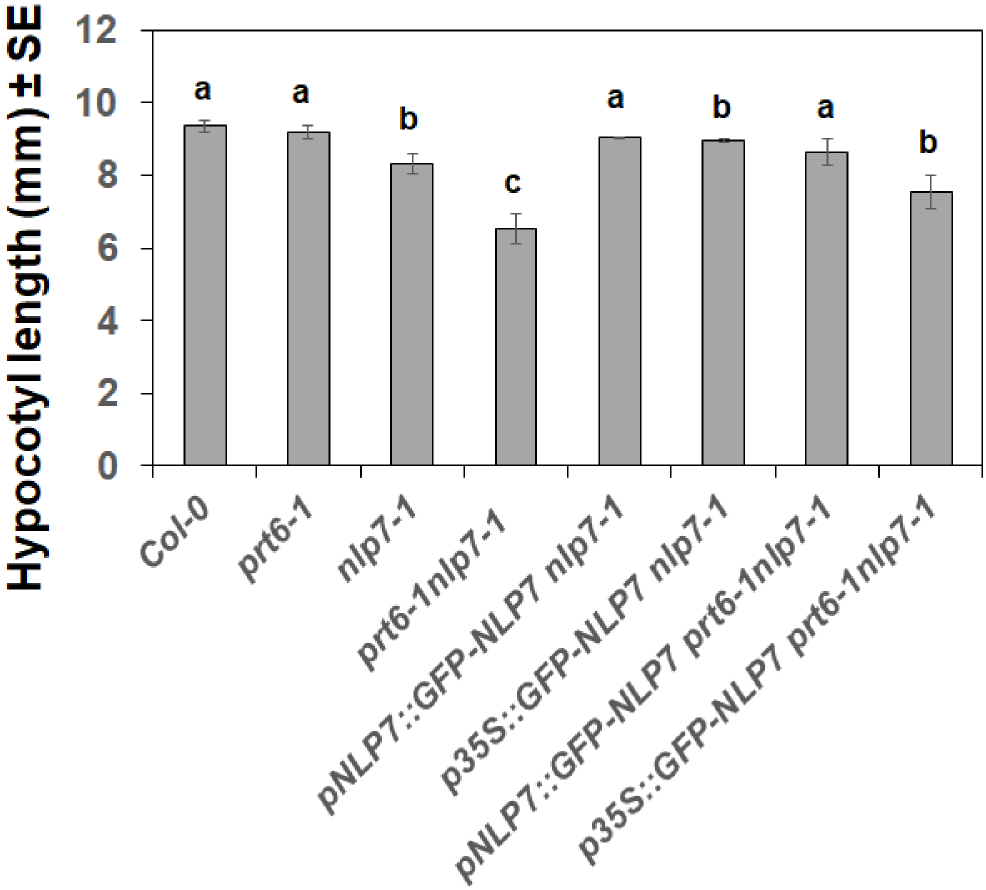

Supplemental Figure S2. Vegetative growth phenotype of mutant and NLP7-overexpressing plants. A, Images of 4-week-old seedlings of the indicated genotypes grown under long-day-photoperiodic conditions. B, Hypocotyl length of etiolated plants of the indicated genotypes. Values represent the mean ± SE of three independent replicates with 20-25 etiolated seedlings per genotype. Statistical significance was calculated by one-way Anova followed by Tukey´s HSD test for multicomparisons. The letters indicate significant differences (p-value < 0.05).

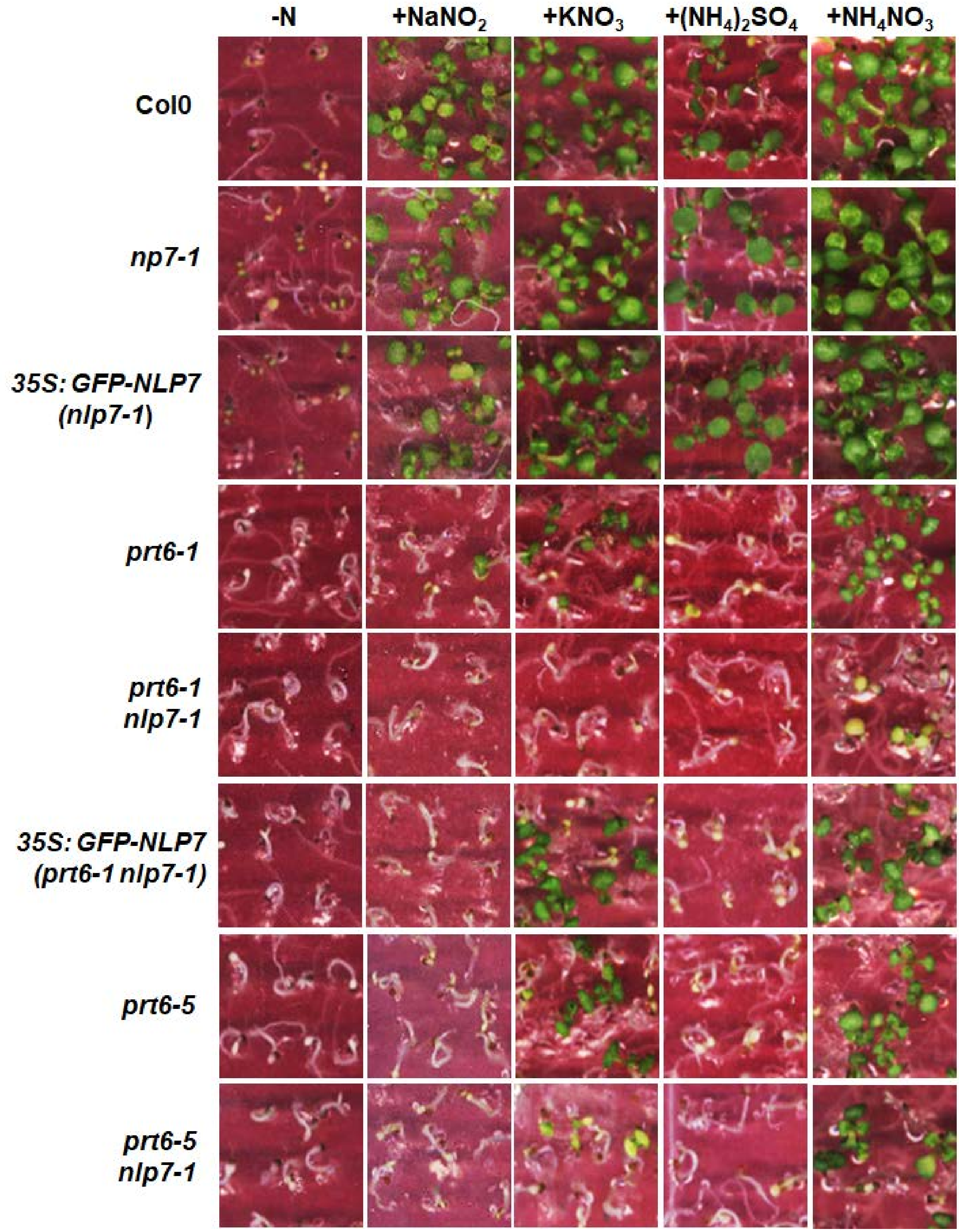

Supplemental Figure S3. Seedling establishment in high sucrose concentrations. Seedling establishment of the indicated wild type and mutant genotypes after sowing seeds in MS medium without N containing 4% sucrose (-N), or that medium supplemented with 5 mM nitrite (+$NaNO_2$), nitrate (+KNO_3), ammonium nitrate (+NH_4NO_3), or 2.5 mM ammonium sulfate (+$(NH_4)_2SO_4$). Plates were phtographed at day 12 after sowing and growing under long-day phtoperiodic conditions.

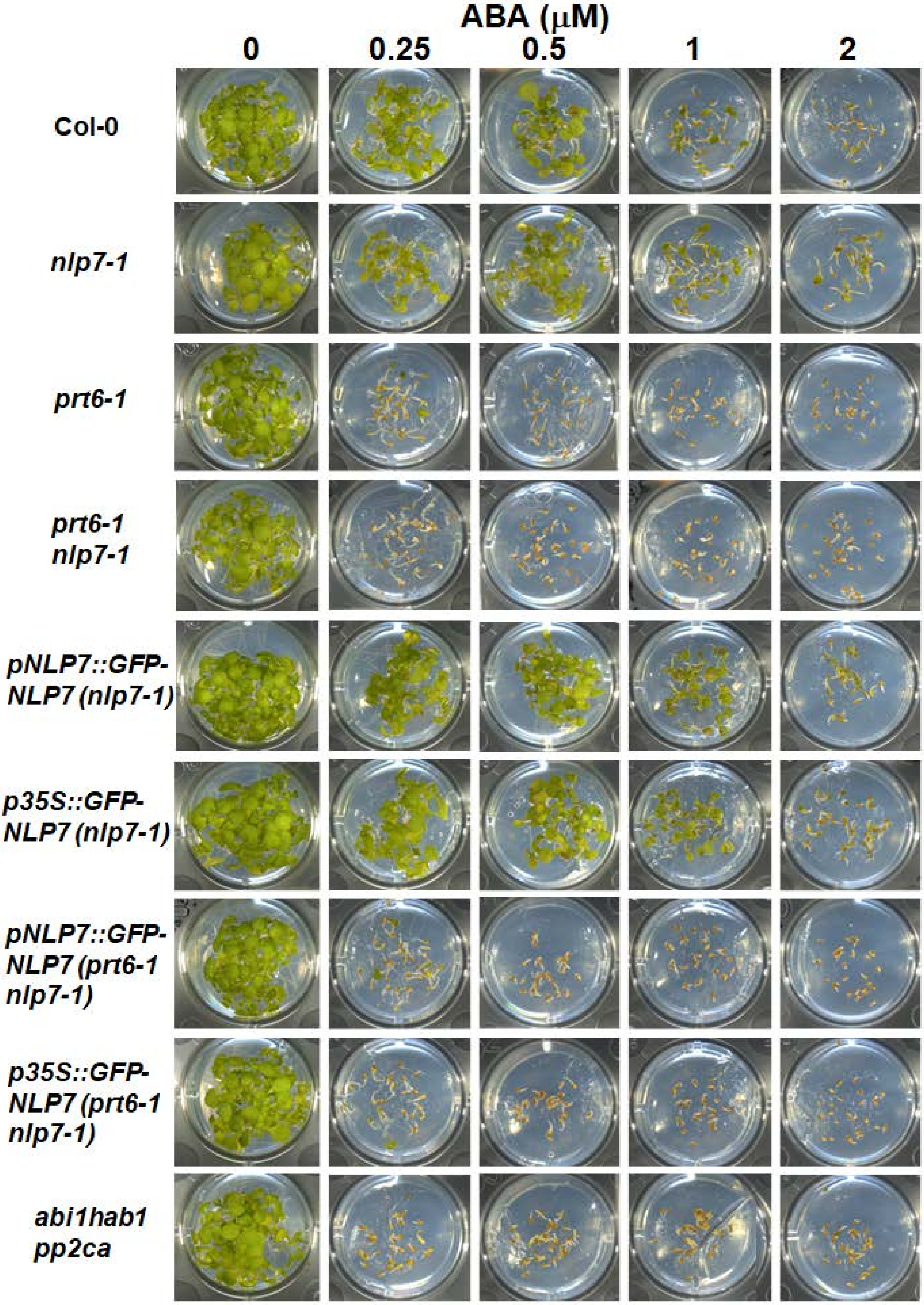

Supplemental Figure S4. Effect of abscisic acid (ABA) on seedling establishment. Seedling establishment of the indicated wild type and mutant or transgenic genotypes after sowing seeds in Murashige-Skoog (MS) medium supplemented with the indicated ABA concentrations. Plates were photographed at day 7 after sowing and growing under long-day photoperiodic conditions.

A

```
MCEPDDNSARNGVTTQPSRSRELLMDVDDLDLDGSWPLDQIPYLSSSNRMISPIFVSSSSEQPC
SPLWAFSDGGGNGFHHATSGGDDEKISSVSGVPSFRLAEYPLFLPYSSPSAAENTTEKHNSFQF
PSPLMSLVPPENTDNYCVIKERMTQALRYFKESTEQHVLAQVWAPVRKNGRDLLTTLGQPFVLN
PNGNGLNQYRMISLTYMFSVDSESDVELGLPGRVFRQKLPEWTPNVQYYSSKEFSRLDHALHYN
VRGTLALPVFNPSGQSCIGVVELIMTSEKIHYAPEVDKVCKALEAVNLKSSEILDHQTTQICNE
SRQNALAEILEVLTVVCETHNLPLAQTWVPCQHGSVLANGGGLKKNCTSFDGSCMGQICMSTTD
MACYVVDAHVWGFRDACLEHHLQKGQGVAGRAFLNGGSCFCRDITKFCKTQYPLVHYALMFKLT
TCFAISLQSSYTGDDSYILEFFLPSSITDDQEQDLLLGSILVTMKEHFQSLRVASGVDFGEDDD
KLSFEIIQALPDKKVHSKIESIRVPFSGFKSNATETMLIPQPVVQSSDPVNEKINVATVNGVVK
EKKKTEKKRGKTEKTISLDVLQQYFTGSLKDAAKSLGVCPTTMKRICRQHGISRWPSRKIKKVN
RSITKLKRVIESVQGTDGGLDLTSMAVSSIPWTHGQTSAQPLNSPNGSKPPELPNTNNSPNHWS
SDHSPNEPNGSPELPPSNGHKRSRTVDESAGTPTSHGSCDGNQLDEPKVPNQDPLFTVGGSPGL
LFPPYSRDHDVSAASFAMPNRLLGSIDHFRGMLIEDAGSSKDLRNLCPTAAFDDKFQDTNWMNN
DNNSNNNLYAPPKEEAIANVACEPSGSEMRTVTIKASYKDDIIRFRISSGSGIMELKDEVAKRL
KVDAGTFDIKYLDDDNEWVLIACDADLQECLEIPRSSRTKIVRLLVHDVTTNLGSSCESTGEL
```

B

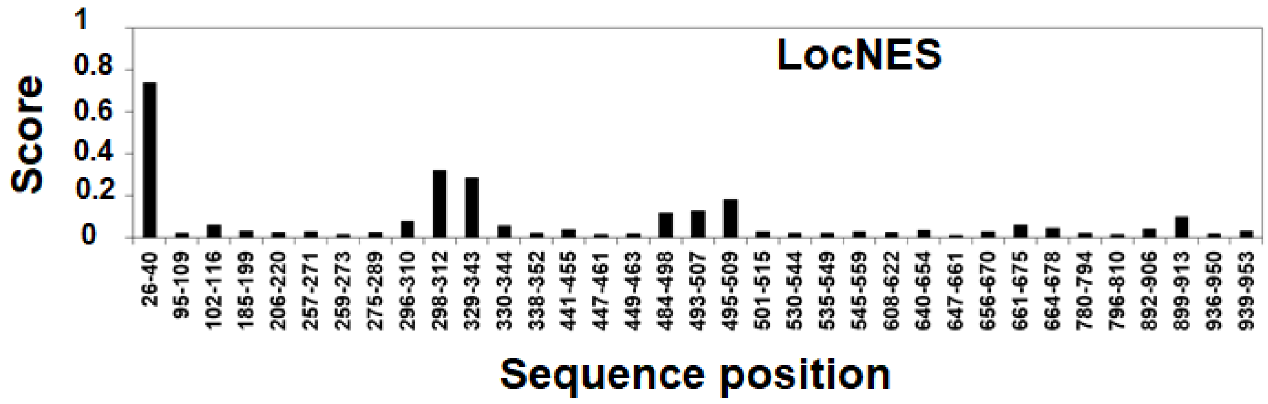

C

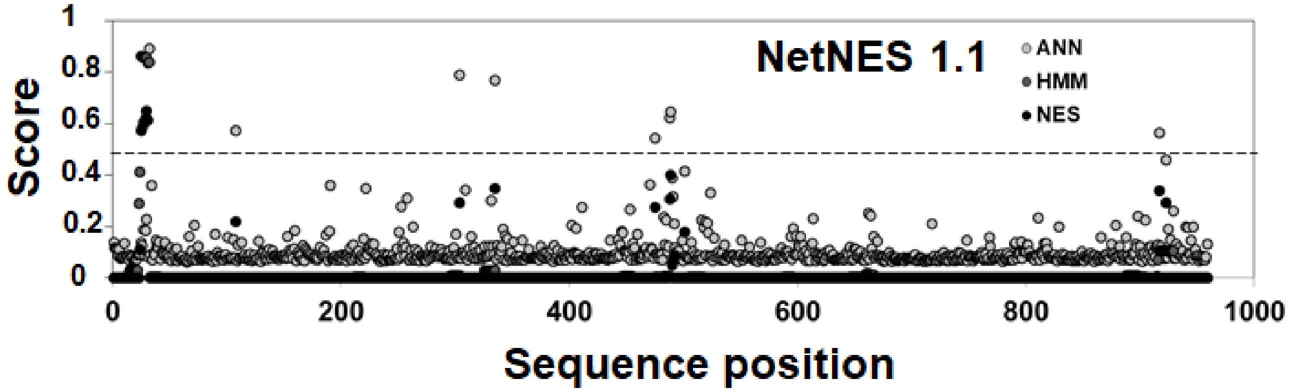

Supplemental Figure S5. Nuclear export signal (NES) in the NLP7 protein. A, Amino acid sequence of the NLP7 protein showing predicted NES highlighted in gray. B, and C, Prediction of NES in the NLP7 protein was performed with LocNES tools (http://prodata.swmed.edu/LRNes/predictNES/) or with NetNES1.1 (http://www.cbs.dtu.dk/), respectively. NES score was calculated from the hidden Markov models (HMM) and Artificial Neural Network (ANN) data.

A

MCEPDDNSARNGVTTQPSRSRELLMDVDDLDLDGSWPLDQIPYLSSSNRMI
SPIFVSSSSEQPCSPLWAFSDGGGNGFHHATSGGDDEKISSVSGVPSFRLA
EYPLFLPYSSPSAAENTTEKHNSFQFPSPLMSLVPPENTDNYCVIKERMTQ
ALRYFKESTEQHVLAQVWAPVRKNGRDLLTTLGQPFVLNPNGNGLNQYRMI
SLTYMFSVDSESDVELGLPGRVFRQKLPEWTPNVQYYSSKEFSRLDHALHY
NVRGTLALPVFNPSGQSCIGVVELIMTSEKIHYAPEVDKVCKALEAVNLKS
SEILDHQTTQICNESRQNALAEILEVLTVVCETHNLPLAQTWVPCQHGSVL
ANGGGLKKNCTSFDGSCMGQICMSTTDMACYVVDAHVWGFRDACLEHHLQK
GQGVAGRAFLNGGSCFCRDITKFCKTQYPLVHYALMFKLTTCFAISLQSSY
TGDDSYILEFFLPSSITDDQEQDLLLGSILVTMKEHFQSLRVASGVDFGED
DDKLSFEIIQALPDKKVHSKIESIRVPFSGFKSNATETMLIPQPVVQSSDP
VNEKINVATVNGVVKEKKKTEKKRGKTEKTISLDVLQQYFTGSLKDAAKSL
GVCPTTMKRICRQHGISRWPSRKIKKVNRSITKLKRVIESVQGTDGGLDLT
SMAVSSIPWTHGQTSAQPLNSPNGSKPPELPNTNNSPNHWSSDHSPNEPNG
SPELPPSNGHKRSRTVDESAGTPTSHGSCDGNQLDEPKVPNQDPLFTVGGS
PGLLFPPYSRDHDVSAASFAMPNRLLGSIDHFRGMLIEDAGSSKDLRNLCP
TAAFDDKFQDTNWMNNDNNSNNNLYAPPKEEAIANVACEPSGSEMRTVTIK
ASYKDDIIRFRISSGSGIMELKDEVAKRLKVDAGTFDIKYLDDDNEWVLIA
CDADLQECLEIPRSSRTKIVRLLVHDVTTNLGSSCESTGEL

B

Position	Peptide	Score	Cutoff	P-value
845	NNLYAPPKEEAIANV	9.177	3.24	0.023
889	GSGIMELKDEVAKRL	12.115	3.24	0.006

C

Position	Peptide	Score	Cutoff
157	RMTQALRYFKESTEQ	1,463	1,16
288	MTSEKIHYAPEVDKV	1,363	1,16
2	******MCEPDDNSA	22,65	20,743
374	CTSFDGSCMGQICMS	2,821	2,454

D

Position	Sequence motif	Potential	Jury agreement	Glycosylation
118	NTTE	0.6080	(6/9)	+
267	NPSG	0.5000	(4/9)	+
319	NESR	0.6168	(7/9)	+
366	NCTS	0.7037	(9/9)	++
544	NATE	0.5733	(6/9)	+
640	NRSI	0.5828	(8/9)	+
686	NGSK	0.5114	(4/9)	+

Supplemental Figure S6. Predicted post-translational modifications (PTMs) in the NLP7 protein. A, Amino acid sequence of the NLP7 protein showing the RWP-RK and PB1 domains and predicted (highligthed in yellow) SUMOylated Lys (K in green), nitrated Tyr (Y in red), S-nitrosated Cys (C in blue) and N-glycosylated Asn (N in purple). B, and C, Prediction of SUMOylation, nitration and S-nitrosation were performed by using the tools from the Cuckoo Group (http://www.biocuckaoo.org/). D, N-glycosylation prediction was performed by using NetNGlyc 1.0 (http://www.cbs.dtu.dk/services/NetNGlyc/) with intermediate (+) and high (++) confidence.

Supplemental Table S1. Genes targeted by NLP7 and differentially expressed (DEGs) in *prt6-1* plants.

AGI loci	Annotation
AT5G45340	CYP707A3__cytochrome P450, family 707, subfamily A, polypeptide 3
AT1G03840	IDD3_MGP__C2H2 and C2HC zinc fingers superfamily protein
AT3G02550	LBD41__LOB domain-containing protein 41
AT1G21000	PLATZ transcription factor family protein
AT2G44080	ARL__ARGOS-like
AT3G14060	Unknown protein
AT3G02910	AIG2-like (avirulence induced gene) family protein
AT4G39800	ATIPS1_ATMIPS1_ myo-inositol-1-phosphate synthase 1
AT5G03290	IDH-V__isocitrate dehydrogenase V
AT3G19030	Unknown protein
AT2G14750	AKN1_APK_APK1_ATAKN1__APS kinase
AT2G44490	BGLU26_PEN2__Glycosyl hydrolase superfamily protein
AT3G15210	ATERF-4_ERF4/RAP2.5 ethylene responsive element binding factor 4
AT5G40850	AtUPM1_UPM1__urophorphyrin methylase 1
AT4G09420	Disease resistance protein (TIR-NBS class)
AT5G67450	AZF1_ZF1__zinc-finger protein 1
AT5G58900	DIV1__Homeodomain-like transcriptional regulator
AT1G13260	AtRAV1_EDF4_RAV1__related to ABI3/VP1 1
AT1G69310	ATWRKY57_WRKY57__WRKY DNA-binding protein 57
AT4G31800	ATWRKY18_WRKY18__WRKY DNA-binding protein 18
AT2G42280	AKS3_FBH4__basic helix-loop-helix DNA-binding superfamily protein
AT4G35270	NLP2__Plant regulator RWP-RK family protein
AT5G47110	LIL3:2__Chlorophyll A-B binding family protein
AT3G56050	Protein kinase family protein
AT1G15670	KFB01_KMD2__Galactose oxidase/kelch repeat superfamily protein
AT2G30520	RPT2__Phototropic-responsive NPH3 family protein
AT1G78020	FLZ6__Protein of unknown function (DUF581)
AT2G38170	ATCAX1_CAX1_RCI4__cation exchanger 1
AT3G17520	Late embryogenesis abundant protein (LEA) family protein
AT3G52710	Unknown protein

Supplemental Table S2. In silico analysis of Lys ubiquitylation for the NLP7 protein.

Position	Sequence	Ubiquitylation	Score
89	HHATSGGDDEKISSVSGVPSF	N	0.44
**122**	**SPSAAENTTEKHNSFQFPSPL**	**Y**	**0.74**
**148**	**PENTDNYCVIKERMTQALRYF**	**Y**	**0.71**
159	ERMTQALRYFKESTEQHVLAQ	N	0.50
176	VLAQVWAPVRKNGRDLLTTLG	N	0.42
**230**	**LGLPGRVFRQKLPEWTPNVQY**	**Y**	**0.51**
**244**	**WTPNVQYYSSKEFSRLDHALH**	**Y**	**0.55**
285	GVVELIMTSEKIHYAPEVDKV	N	0.31
294	EKIHYAPEVDKVCKALEAVNL	N	0.33
297	HYAPEVDKVCKALEAVNLKSS	N	0.48
305	VCKALEAVNLKSSEILDHQTT	N	0.38
**364**	**GSVLANGGGLKKNCTSFDGSC**	**Y**	**0.54**
365	SVLANGGGLKKNCTSFDGSCM	N	0.46
**408**	**RDACLEHHLQKGQGVAGRAFL**	**Y**	**0.55**
430	GGSCFCRDITKFCKTQYPLVH	N	0.39
433	CFCRDITKFCKTQYPLVHYAL	N	0.27
446	YPLVHYALMFKLTTCFAISLQ	N	0.32
493	LLLGSILVTMKEHFQSLRVAS	N	0.22
**513**	**SGVDFGEDDDKLSFEIIQALP**	**Y**	**0.83**
525	SFEIIQALPDKKVHSKIESIR	N	0.38
526	FEIIQALPDKKVHSKIESIRV	N	0.35
530	QALPDKKVHSKIESIRVPFSG	N	0.38
**542**	**ESIRVPFSGFKSNATETMLIP**	**Y**	**0.52**
565	VVQSSDPVNEKINVATVNGVV	N	0.49
576	INVATVNGVVKEKKKTEKKRG	N	0.28
578	VATVNGVVKEKKKTEKKRGKT	N	0.24
579	ATVNGVVKEKKKTEKKRGKTE	N	0.23
580	TVNGVVKEKKKTEKKRGKTEK	N	0.20
583	GVVKEKKKTEKKRGKTEKTIS	N	0.18
584	VVKEKKKTEKKRGKTEKTISL	N	0.27
587	EKKKTEKKRGKTEKTISLDVL	N	0.25
590	KTEKKRGKTEKTISLDVLQQY	N	0.39
**606**	**VLQQYFTGSLKDAAKSLGVCP**	**Y**	**0.67**
**610**	**YFTGSLKDAAKSLGVCPTTMK**	**Y**	**0.63**
620	KSLGVCPTTMKRICRQHGISR	N	0.35
635	QHGISRWPSRKIKKVNRSITK	N	0.27
637	GISRWPSRKIKKVNRSITKLK	N	0.12

638	ISRWPSRKIKKVNRSITKLKR	N	0.33
645	KIKKVNRSITKLKRVIESVQG	N	0.13
647	KKVNRSITKLKRVIESVQGTD	N	0.16
689	AQPLNSPNGSKPPELPNTNNS	N	0.39
725	SPELPPSNGHKRSRTVDESAG	N	0.36
752	SCDGNQLDEPKVPNQDPLFTV	N	0.42
**809**	**GMLIEDAGSSKDLRNLCPTAA**	**Y**	**0.90**
823	NLCPTAAFDDKFQDTNWMNND	N	0.48
845	NSNNNLYAPPKEEAIANVACE	N	0.48
867	SGSEMRTVTIKASYKDDIIRF	N	0.32
871	MRTVTIKASYKDDIIRFRISS	N	0.13
**889**	**ISSGSGIMELKDEVAKRLKVD**	**Y**	**0.64**
**894**	**GIMELKDEVAKRLKVDAGTFD**	**Y**	**0.55**
897	ELKDEVAKRLKVDAGTFDIKY	N	0.25
906	LKVDAGTFDIKYLDDDNEWVL	N	0.20
936	CLEIPRSSRTKIVRLLVHDVT	N	0.17

Supplemental Table S3. Oligonucleotides used in this work.

Name	Sequence (5´to 3´)	AGI	Application
NLP7-F	ATGTGCGAGCCCGATGATAATTCCGC	AT4G24020	Cloning *NLP7*
nostop-NLP7-R	CAATTCTCCAGTGCTCTCGCAGG	AT4G24020	Cloning *NLP7*
SeqNLP7_2R	GGTTAGGTGAATTATTGGTG	AT4G24020	Sequencing *NLP7*
SeqNLP7_1F	ATAGTGAAAGTGACGTAGAG	AT4G24020	Sequencing *NLP7*
SeqNLP7_3R	CGGTTCATCTAATTGGTTAC	AT4G24020	Sequencing *NLP7*
LBb1.3	ATTTTGCCGATTTCGGAAC		Genotyping SALK
SALK_026134C_LP	AAGAATCAACCGAACAACACG	AT4G24020	Genotyping *nlp7-1*
SALK_026134C_RP	CTTCAAAATAGCAGGCCAAATG	AT4G24020	Genotyping *nlp7-1*
LB2	CCAAACTGGAACAACACTCAACCCTATCTC		Genotyping *prt6-5*
At120	AAAATTGATCCTTTCCATGCC	AT5G02310	Genotyping *prt6-5*
At121	CAACATAAGAATCTGCGGGAG	AT5G02310	Genotyping *prt6-1*
LB_SAIL	GCTTCCTATTATATCTTCCCAAATTACCAATACA	AT5G02310	Genotyping *prt6-1*
qNIA1-F	AGGTTTGGAAGGCGAATCG	AT1G77760	qRT-PCR
qNIA1-R	TGGCTGCAACGCAAACTG	AT1G77760	qRT-PCR
qNIA2-F	CCCGTTGCACTACGTTCGTA	AT1G37130	qRT-PCR
qNIA2-R	CGTCCATTCGGCCCATT	AT1G37130	qRT-PCR
qNLP7-F	GAGTTTGCCCGACGACAATGAAG	AT4G24020	qRT-PCR
qNLP7-R	GGCCTCCATCAGTACCTTGAACAG	AT4G24020	qRT-PCR
qACT2-F	TTGTTCCAGCCCTCGTTTGT	AT3G18780	qRT-PCR
qACT2-R	TGTCTCGTGGATTCCAGCAG	AT3G18780	qRT-PCR
